# Cytotoxic conjugates of betulinic acid and substituted triazoles prepared by Huisgen Cycloaddition from 30-azidoderivatives

**DOI:** 10.1371/journal.pone.0171621

**Published:** 2017-02-03

**Authors:** Veronika Sidova, Pavel Zoufaly, Jan Pokorny, Petr Dzubak, Marian Hajduch, Igor Popa, Milan Urban

**Affiliations:** 1 Department of Organic Chemistry, Faculty of Science, Palacky University, Olomouc, Czech Republic; 2 Institute of Molecular and Translational Medicine, Faculty of Medicine and Dentistry, Palacky University, Olomouc, Czech Republic; National Cancer Institute at Frederick, UNITED STATES

## Abstract

In this work, we describe synthesis of conjugates of betulinic acid with substituted triazoles prepared *via* Huisgen 1,3-cycloaddition. All compounds contain free 28-COOH group. Allylic bromination of protected betulinic acid by NBS gave corresponding 30-bromoderivatives, their substitution with sodium azides produced 30-azidoderivatives and these azides were subjected to Cu^I^ catalysed Huisgen 1,3-cycloaddition to give the final conjugates. Reactions had moderate to high yields. All new compounds were tested for their *in vitro* cytotoxic activities on eight cancer and two non-cancer cell lines. The most active compounds were conjugates of 3β-*O*-acetylbetulinic acid and among them, conjugate with triazole substituted by benzaldehyde **9b** was the best with IC_50_ of 3.3 μM and therapeutic index of 9.1. Five compounds in this study had IC_50_ below 10 μM and inhibited DNA and RNA synthesis and caused block in G0/G1 cell cycle phase which is highly similar to actinomycin D. It is unusual that here prepared 3β-*O*-acetates were more active than compounds with the free 3-OH group and this suggests that this set may have common mechanism of action that is different from the mechanism of action of previously known 3β-*O*-acetoxybetulinic acid derivatives. Benzaldehyde type conjugate **9b** is the best candidate for further drug development.

## Introduction

Triterpenes are natural compounds that may be found in almost all living organisms and they are particularly prevalent in plants [1]. These compounds are not part of the main metabolic pathways, they are secondary metabolites. Interestingly, they have a variety of biological activities, which may be the reason why organisms produce them. Among the activities, we may find antitumor, antibacterial, anticariogenic, antiparasitic, antifungal and many others [2–11]. In our research group we are developing new derivatives of betulinic acid (**1**) in order to find compounds with higher cytotoxicity and better pharmacological properties than the parent compound. One of the possibilities explored was annealing of a heterocycle to the main terpenic skeleton, which resulted in a small library of about fifty new compounds [12–15]. Among them, four derivatives had IC_50_ in low micromolar range and currently belong to our most promising compounds in *in vivo* tests. All four active heterocycles are derivatives of betulinic acid (**1**). Recently, a number of new triterpenoid heterocycles were prepared and a number of them had high cytotoxic activity [16–20]. To improve pharmacological properties of triterpenes, especially their solubility in water, various modifications of triterpenes were done and some of them were successful, especially compounds with another (polar) molecule connected to them. Examples include esters with sugars, glycosides, esters with dicarboxylic acids, conjugates with polyethylene glycol, ammonium salts etc [21–29].

Recently, a number of new articles were published on connecting a terpene with another molecule of interest *via* Huisgen 1,3-cycloaddition reaction. The first approach used terpenes substituted with alkynes in the position 3 [30–34], second approach used propargylesters or amides prepared at 28-COOH group [35–45], and the third approach used 30-azidoderivatives prepared *via* 30-bromoderivatives [46;47]. Rarely, also position 2 is modified [48] or two position at once (3 and 30) in [49].

In this work, we decided to explore the third option and to connect betulinic acid (**1**) to other molecules of interest. Introduction of a rather polar triazole ring capable of forming hydrogen bonds was expected to improve solubility of the target molecules in water based media and bioavailability [50;51]. Possibly, the triazole ring may also become a part of the pharmacophore. On the other hand, introduction of a completely new moiety on the other side of the triazole ring (another aromatic rings—both heterocyclic and carbocyclic, aldehydes, amines etc.) could change the biological properties such as cytotoxicity or selectivity and the new compounds could act by different mechanism of action than the parent betulinic acid (**1**). Among the derivatives prepared by other research groups there are only few examples [46;47] containing both free 28-carboxylic group and free 3-hydroxy group. In most cases, cycloaddition reactions were done with acid **1** protected as methyl ester or as acetate and the final molecules were also tested with the protective group on. There are many examples in the literature [21;52;53] that betulinic acid (**1**) derivatives are highly cytotoxic when unprotected while methylesters and acetates are usually inactive. Therefore, the main aim of this work was to explore unprotected derivatives of betulinic acid (**1**) modified by cycloaddition reactions in the position 30 and to explore their cytotoxic activity and influence on cancer cells.

## Results and discussion

### Chemistry

**Fig 1:**
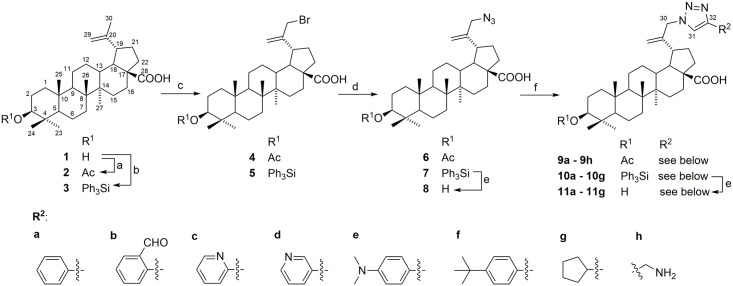
The preparation of all derivatives. Reagents and conditions: (a) Ac_2_O, pyridine, r.t. 16 h; (b) Ph_3_SiCl, DMF, imidazole, r.t. 36 h; (c) NBS, AIBN, CCl_4_, 75°C for 1 h then 50°C for 3 h; (d) NaN_3_, DMSO, r.t., 36 h; (e) TBAF, THF, r.t., 18–32 h, or HCl, CH_2_Cl_2_, r.t., 5–11 h; (f) azides **6**, **7** or **8**, CuSO_4_·5H_2_O, sodium L-ascorbate, alkynes.

Bromination of betulinic acid (**1**) derivatives at the allylic position (C-30) is described in the literature [54–57] that mostly used NBS as the bromination agent, AIBN as a radical source and CCl_4_ as an inert solvent. In this work, we found that the method afforded only low yields when free acid **1** was used. All reagents have limited solubility in CCl_4_ and dibromoderivative starts forming before the full conversion of the starting betulinic acid (**1**) to 30-bromobetulinic acid. To increase the solubility of acid **1**, we choose to protect it at the position 3 as acetate or triphenylsilylether. We decided to leave the 28-COOH group unprotected, since this neopentyl-type ester requires harsh conditions for its deprotection and the presence of free carboxylic group should not interfere with the following reactions. For protection of 3β-OH group, acetate was chosen as a stable protective group that would be cleavable in basic conditions, triphenylsilyl group was chosen as more labile protective group easily removable in acidic conditions or by the fluorine anion. This should allow for almost unlimited variability of the new substituents. Both acetate **2** and triphenylsilyl derivative **3** were synthesized by standard procedures. Bromination of **2** and **3** afforded good yields of pure bromoderivatives **4** and **5**. The reaction of bromoderivatives **4** and **5** with sodium azide gave corresponding 30-azidoderivatives **7**, **8**, and to obtain the unprotected azide **6**, silylether **8** was deprotected by TBAF in THF; Fig 1.

Final derivatives containing acetate or triphenylsilylether at the position 3 (compounds **9a**–**9h**, **10a**–**10g**) were prepared by Huisgen 1,3-cycloaddition from azides **6** and **7**. Depending on the reactivity, one or two equivalents of alkyne was used and the reaction was performed either at r.t. or at 50°C. Yields were moderate to high with the only exception—reaction of each azide **6**–**8** with propargylamine that gave 10% yield of acetate **9h** but no sililated nor free product was obtained. Reaction with FMOC or BOC protected propargylamine also did not yield the desired product. In all cases, a mixture of polar unseparable compounds was obtained and we were unable to isolate the desired product. The cycloaddion was catalyzed by Cu^I^ species which ensured that only the proposed 1,4-isomer formed. Traces of Cu were removed by treatment of the crude products with H_2_S before chromatography but the amount of remaining metal was not determined. This will be performed if any compound of this set will enter further biological tests. Free derivatives **11a**–**11g** were obtained by the deprotection of their silylated analogues using TBAF in THF (all **11a**–**11g** were prepared this way) with reasonable yields 58–81%. To find out if the compounds are stable during acidic deprotection procedure, derivatives **10a**, **10c**, **10d**, and **10f** were also deprotected by HCl in CHCl_3_ with comparable yields of 62–76%. Attempts to prepare compounds **11c** and **11d** directly from the unprotected azide **8** were also successful with average yields of 75%. We may conclude, that all three ways to the unprotected derivatives **11a**–**11g** give similar results.

### Biological assay

#### Cytotoxicity

Cytotoxic activity of all synthesized compounds was investigated *in vitro* against eight human cancer cell lines and two non-tumor fibroblasts using the standard MTS test (Table 1). The cancer cell lines were derived from T-lymphoblastic leukemia CCRF-CEM, leukemia K562 and their multiresistant counterparts expressing P-glycoprotein, MRP1 and LRP proteins (CEM-DNR, K562-TAX) [58], solid tumors including lung (A549) and colon (HCT116, HCT116p53-/-) carcinomas, osteosarcoma cell line (U2OS), and for comparison, tests were performed on two human non-cancer fibroblast cell lines (BJ, MRC-5).

**Table 1:** Cytotoxic activities of prepared derivatives on eight tumor (including resistant) and two normal fibroblast cell lines. All other compounds prepared in this work were also tested but their activities on these 10 cell lines were higher than 50 μM which is considered inactive.

Comp.	IC_50_ (μM/L)^a^										
	CCRF-CEM	CEM-DNR	K562	K562- TAX	A549	HCT116	HCT116p53^-/-^	U2OS	BJ	MRC-5	TI^b^
**1**	45.5	45.4	40.0	43.1	43.4	38.0	>50.0	>50.0	37.6	32.9	0.7
**4**	5.7	15.3	21.7	15.8	13.2	15.9	14.1	21.2	31.7	24.2	4.9
**5**	>50.0	13.2	>50.0	12.2	>50.0	35.6	31.1	27.9	>50.0	>50.0	-
**6**	7.4	19.2	12.3	15.5	17.6	18.6	19.7	20.9	26.7	20.0	3.2
**8**	21.7	25.8	17.2	23.9	23.0	25.5	24.0	25.0	34.0	19.1	1.2
**9a**	13.4	19.5	15.1	19.1	23.4	25.1	31.1	24.8	>50.0	32.4	>3.1
**9b**	3.3	4.0	3.6	3.9	14.8	6.4	9.5	12.8	31.3	28.9	9.1
**9c**	9.0	14.4	22.1	13.8	13.2	30.3	13.7	16.0	29.5	27.6	3.2
**9d**	14.9	12.0	13.3	11.2	11.3	19.2	18.7	17.3	29.9	28.2	1.9
**9e**	19.4	29.6	35.4	30.0	26.6	32.9	33.1	31.0	35.1	30.3	1.6
**9f**	34.5	15.0	15.6	23.4	45.0	41.9	48.4	45.8	>50.0	>50.0	>1.4
**9g**	26.2	30.8	43.6	29.1	>50.0	38.3	47.6	44.1	>50.0	>50.0	>1.9
**9h**	20.2	28.3	>50.0	>50.0	30.5	32.0	>50.0	30.6	>50.0	>50.0	>2.5
**11a**	16.6	23.8	>50.0	23.3	>50.0	>50.0	>50.0	>50.0	>50.0	>50.0	>3.0
**11b**	8.5	11.5	>50.0	13.7	>50.0	26.3	26.9	>50.0	>50.0	>50.0	>5.9
**11c**	14.4	8.5	>50.0	7.2	>50.0	>50.0	>50.0	>50.0	>50.0	>50.0	>3.5
**11f**	>50.0	13.7	>50.0	16.0	>50.0	36.7	>50.0	>50.0	>50.0	>50.0	-
**11g**	16.7	38.0	>50.0	23.3	>50.0	33.5	>50.0	>50.0	>50.0	>50.0	>3.0

^a^The lowest concentration that kills 50% of cells. The standard deviation in cytotoxicity assays is typically up to 15% of the average value. Compounds with IC_50_ > 50 μM are considered inactive.

^b^Therapeutic index is calculated for IC_50_ of CCRF-CEM line vs average of both fibroblasts.

All derivatives prepared within this study have free 28-COOH group that was expected to be essential for retaining of the biological activity. Cytotoxicity of the selected starting compounds and also compounds modified at C-30 by Huisgen 1,3-cycloadditions are in Table 1 (acetylated derivatives **9a**–**9h**, silylated analogues **10a**–**10g**, and fully deprotected derivatives **11a**–**11g** that were expected to be the most active).

Among the starting material, bromide **4** and azide **6** had significant activity (IC_50_ 5.7 and 7.4 μM) on multiple cancer cell lines with therapeutic index of 4.9 or 3.2 (calculated for the reference CCRF-CEM line). To our surprise, both compounds **4** and **6** are 3β-*O*-acetates, which is in contrast to our initial assumptions that acetates should be less active than compounds with the free 3β-hydroxy group. In addition, our results indicate, that acetates **9a**–**9h** are often highly active (with IC_50_ in low micromolar ranges) on multiple cancer cell lines (parental and mutiresistant) and in most cases, they are more active than their non-acetylated analogues **11a**–**11g**. The most active compound of this study is derivative **9b** (IC_50_ 3.3 μM on the reference CCRF-CEM cell line) which belongs among acetates and contains benzaldehyde connected to the position 30 through the triazole ring formed by the cycloaddition. The compound has reasonable therapeutic index 9.1 and seems the most promising derivative of this study. Its non-acetylated derivative is also active on the reference line (IC_50_ 8.5 μM) and we see this trend throughout all of the prepared derivatives, compounds **9a**–**9g** are more active than free compounds **11a**–**11g** with only few exceptions.

In general, it seems that modified C-30 position, conjugated to a large triazole-aromatic substituent became an important part of the pharmacophore and is responsible for the cytotoxicity. In contrast, the functional group at C-3 probably influences the bioavailability of each molecule. Compounds with free both 28-COOH and 3β-OH groups contain two hydrophilic functional group on the opposite sides of their molecules and this may interfere with their permeability through cellular membranes. Small and lipophilic acetate on one side of the molecule can solve this problem.

#### Cell cycle analysis

We have observed that most cytotoxic derivatives from this study are 3*O*-acetylated 30-bromo, 30-azido derivatives 4 and 6, 3*O*-acetylated conjugates 9b and 9c, and one 3-hydroxyderivative 11b. All of them are inhibiting DNA and RNA synthesis. The inhibition of the cell cycle in G0/G1 was observed with highest accumulation after treatment with acetylated derivative 9b. The high percentage of apoptotic cells (sub G1) is observed at 5 × IC_50_ concentration, pointing on rapid induction of apoptosis (Table 2).

**Table 2:** Influence of compounds 4, 6, 9b, 9c, 11b on cell cycle, DNA and RNA synthesis at 1^a^ × and 5^b^ × IC_50_.

Comp.	Used conc. (μM)	Sub G1 (%)	G0/G1 (%)	S (%)	G2/M (%)	pH3^Ser10^ (%)	DNA synthesis	RNA synthesis
**Control**	0	2.2	38.4	42.4	19.3	2.1	37.5	42.1
**4**	5.7^a^	9.4	47.3	35.9	16.8	1.34	38.6	56.3
**4**	28.5^b^	39.7	33.7	43.0	23.2	2.35	5.80	24.8
**6**	7.4^a^	10.7	49.3	30.4	20.3	1.80	33.0	14.1
**6**	37.0^b^	70.0	41.4	34.8	23.8	2.68	1.00	1.70
**9b**	3.3^a^	10.8	58.6	21.9	19.5	1.19	22.6	15.8
**9b**	16.5^b^	51.9	35.7	46.2	18.1	0.37	0.55	0.08
**9c**	9.0^a^	7.2	48.9	30.9	20.3	1.47	20.3	25.4
**9c**	45.0^b^	65.6	40.5	38.5	21.0	0.89	2.90	0.40
**11b**	8.5^a^	65.6	59.0	25.3	15.7	0.88	20.0	48.3
**11b**	42.5^b^	65.6	45.5	35.4	19.1	1.19	6.40	38.1

^a^The values were obtained at 1 × IC_50_.

^b^The values were obtained at 5 × IC_50_.

## Conclusions

Three sets of betulinic acid derivatives modified at C-30 were prepared by Huisgen 1,3-cycloaddition catalyzed by Cu^I^ species. All compounds have free 28-COOH and the first set are 3β-*O*-acetates **9a**–**9h**, the second set are 3β-silylethers **10a**–**10g**, and the third set are compounds with free 3β-OH group **11a**–**11g**. All compounds were subjected to tests of cytotoxicity on 8 cancer cell lines and 2 non cancer fibroblasts. Several derivatives had IC_50_ in low micromolar ranges for parental and multiresistant cell lines, the best compound was aldehyde-acetate **9b** which also had high therapeutic index and this makes the compound the most promising candidate for future *in vivo* tests and for studies of mechanism of action. In this work, unusual trend was found between the activities of 3β-*O*-acetates vs. free compounds. Acetates **9a**–**9h**, were usually more active than free derivatives **11a**–**11g**. This suggests that compounds prepared in this study may have mechanism of action that differs from acetylated betulinic derivatives known from the literature [21] where this trend is opposite. Moreover, the inhibition of DNA and RNA was observed even at 1 × IC_50_ concentrations together with G1 cell cycle block which is highly similar to actinomycin D behavior [59]. Thus, one may speculate that conjugation of the new triazole ring equipped with carbocyclic (or heterocyclic) ring forms a new type of pharmacophore. To prove it, however, the compound will have to be further transformed into a probe suitable for pull down assays [49] or into a fluorescent probe and more biological tests will have to be done.

## Experimental

### General experimental procedures

#### Materials and instruments

Melting points were determined using a Büchi B-545 apparatus and are uncorrected. Optical rotations were measured on an Autopol III (Rudolph Research, Flanders, USA) polarimeter in MeOH at 25°C unless otherwise stated and are in [10^−1^ deg cm^2^ g^-1^]. ^1^H and ^13^C NMR spectra were recorded on Varian^UNITY^ Inova 400 (400 MHz for ^1^H) or Varian^UNITY^ Inova 300 (300 MHz for ^1^H) or Jeol ECX-500SS (500 MHz for ^1^H) instruments, using CDCl_3_, D_6_-DMSO or CD_3_OD as solvents (25°C). Chemical shifts were eider referenced to the residual signal of the solvent (CDCl_3_, D_6_-DMSO) or to tetramethylsilane added as an internal standard. ^13^C NMR spectra were eider referenced to CDCl_3_ (77.00 ppm) or D_6_-DMSO (39.51 ppm) or to tetramethylsilane added as an internal standard. EI MS spectra were recorded on an INCOS 50 (Finigan MAT) spectrometer at 70 eV and an ion source temperature of 150°C. The samples were introduced from a direct exposure probe at a heating rate of 10 mA/s. Relative abundances stated are related to the most abundant ion in the region of *m*/*z* > 180. HRMS analysis was performed using LC-MS an Orbitrap high-resolution mass spectrometer (Dionex Ultimate 3000, Thermo Exactive plus, MA, USA) operating at positive full scan mode in the range of 100–1000 m/z. The settings for electrospray ionization were as follows: oven temperature of 150°C, source voltage of 3,6 kV. The acquired data were internally calibrated with phthalate as a contaminant in methanol (m/z 297.15909). Samples were diluted to a final concentration of 0.1 mg/mL in methanol. The samples were injected to mass spectrometer over autosampler after HPLC separation: precolumn phenomenex 2.6 μm C18. Mobile phase isokrat. CH_3_CN/IPA/amonium acetate 0.01M 80/10/10, flow 0,3 mL/min. IR spectra were recorded on a Nicolet Avatar 370 FTIR. DRIFT stands for Diffuse Reflectance Infrared Fourier Transform. TLC was carried out on Kieselgel 60 F254 plates (Merck) detected by spraying with 10% aqueous H_2_SO_4_ and heating to 150–200°C. Starting triterpenes—betulin (1), dihydrobetulonic acid (2b), and allobetulin (3a) were obtained from company Betulinines (www.betulinines.com). All other chemicals and solvents were obtained from Sigma-Aldrich.

### Synthetic procedures

#### General procedure for Huisgen cycloaddition of triterpenic azides

Each azide was dissolved in DMF (4 mL/100 mg) and sodium L-ascorbate (0.5 equiv.) was added followed by CuSO_4_·5H_2_O. The reaction mixture was stirred until its color turned green which is the sign for Cu^I^ species being formed (usually 20 min). Then, each alkyne was added (1–2 equiv.) and the reaction mixture was stirred at room temperature (or 50°C) for various time, conditions are specified for each compound. The reaction was monitored using TLC in hexane/EtOAc in ratios 3: 1–1: 2 depending on substrates. After the reaction was completed, the mixture was poured on ice where the product precipitated. The precipitate was filtered on frit, washed with water and dried in desiccator, then it was dissolved in EtOAc, traces of copper ions were precipitated by H_2_S and filtered off. Product was then purified by column chromatography on silica gel (100 × weight of the terpene) in hexane/EtOAc or cyclohexane/EtOAc in various ratio. Analytical samples were purified on HPLC, crystallized or lyophilized. Specific conditions, such as reaction times, temperature, and mobile phase for TLC, CC or HPLC are specified in each experiment.

#### General procedures for the deprotection of silylated compounds

Procedure 1: each triazole (0.2 mmol) was dissolved in THF (5 mL), then TBAF (2 mL; 10 equiv.; 1M solution in THF) was added. The reaction mixture was stirred at various temperature until the reaction was completed (monitored by TLC with 5% MeOH in CHCl_3_ as mobile phase), the deprotection usually took 18–32 h. The reaction mixture was poured to water and the product was extracted to EtOAc. The organic phase was washed twice with 5% NaHCO_3_ and with water, dried over MgSO_4_ and evaporated. Crude product was chromatographed on silica gel (10–20 g) in gradient CHCl_3_ to 10% MeOH in CHCl_3_. Analytical samples and samples for biological tests were purified on reverse phase C-18 HPLC in isocratic mobile phase: 80% CH_3_CN, 20% buffer (0.1% NH_4_OAc in water). Reaction temperature and time is specified at each experiment.

Procedure 2: each triazole (0.2 mmol) was dissolved in CH_2_Cl_2_ (5 mL) and HCl (0.3 mL, 35% in water) was added. The reaction mixture was stirred at r.t. for 5–11 h while monitored on TLC with 5% MeOH in CHCl_3_ as mobile phase. After the reaction was completed, 5% NaHCO_3_ in water was added to adjust the pH to about 5. The mixture was stirred yet another 1 h, then poured to water, extracted to CHCl_3_, washed with water and dried over MgSO_4_. Organic solvents were evaporated in vacuo and the crude product was chromatographed on silica gel (10–20 g) in gradient CHCl_3_ to 10% MeOH in CHCl_3_. Analytical samples and samples for biological tests were purified on reverse phase C-18 HPLC in isocratic mobile phase: 80% CH_3_CN, 20% buffer (0.1% NH_4_OAc in water).

#### 3β-Triphenylsililbetulinic acid 3

5 g (10.9 mmol) of betulinic acid 1 was dissolved in DMF (100 mL), then Ph_3_SiCl (5.9 g, 20 mmol) and imidazole (1.4 g, 21 mmol) was added. The reaction mixture was stirred at r.t. for 36 h while being monitored on TLC (hexane/EtOAc 4: 1). The crude reaction mixture was poured on ice while the product precipitated, the precipitate was filtered off on a frit, washed with water and dried in desiccator. Crude product was chromatographed on silica gel (200 g) in gradient of hexane/EtOAc from 5: 1 to 2: 1 and crystallized from hexane.

Compound **3** was obtained as white crystals, 6.6 g (85%): mp 147–148°C. IR (DRIFT): 2400–3400, 1720, 1692, 1642 cm^-1^. ^1^H NMR (500 MHz, CDCl_3_): δ 0.84 (s, 3H); 0.88 (s, 3H); 0.92 (s, 3H); 0.93 (s, 3H); 0.96 (s, 3H, H-23, 24, 25, 26, 27); 1.68 (s, 3H, H-30); 2.16 (td, 1H, *J*_1_ = 12.9 Hz, *J*_2_ = 3.7 Hz); 2.27 (dt, 1H, *J*_1_ = 12.9 Hz, *J*_2_ = 3.2 Hz); 3.00 (td, 1H, *J*_1_ = 10.9 Hz, *J*_2_ = 4.6 Hz, H-19β); 3.34 (dd, 1H, *J*_1_ = 11.8 Hz, *J*_2_ = 4.6 Hz, H-3α); 4.60 (dd, 1H, *J*_1_ = 2.0 Hz, *J*_2_ = 1.5 Hz, H-29 *pro-E*), 4.73 (d, 1H, *J* = 2.0 Hz, H-29 *pro-Z*); 7.35–7.41 (m, 6H); 7.42–7.46 (m, 3H); 7.63–7.68 (m, 6H, 15 × H-Ph). ^13^C NMR (125 MHz, CDCl_3_): δ = 14.68; 15.99; 16.12; 16.33; 18.40; 19.35; 20.77; 25.43; 27.92; 28.45; 29.65; 30.65; 32.14; 34.26; 37.00; 38.39; 38.54; 39.57; 40.63; 42.39; 46.89; 49.24; 50.32; 55.24; 56.37; 77.20; 81.16; 109.64; 127.67; 129.69; 135.38; 135.55; 150.37; 182.06. MS (ESI-): *m*/*z* (%) = 713 (100, [M-H]^-^). HRMS (ESI-) *m*/*z* calcd for C_48_H_63_O_3_Si [M-H]^-^ 713.4384, found 713.4373.

#### Bromination of the position C-30 in derivatives 2 and 3

Each derivative 2 and 3 (2.8 mmol) was dissolved in CCl_4_ (30 mL). Then, NBS (0.8 g, 4.5 mmol) and AIBN (0.14 mmol, 5%) was added and the reaction mixture was stirred at 75°C for 1h. Then, the reaction mixture was stirred at 50°C for 3 h and another several hours (TLC) at 5°C to finish the reaction completion. The reaction had to be frequently monitored by TLC in hexane/EtOAc (2: 1 for product 4 and 5: 1 for 5) because keeping the reaction mixture at elevated temperature for longer period than necessary leads to dibrominated sideproducts. After the completion, the reaction mixture was poured into water, extracted to EtOAc, 3 × washed with water, dried over MgSO_4_ and evaporated. Crude products were purified on silica gel in gradient of hexane/EtOAc 4: 1 to 1: 1 and crystallized from hexane.

Compound **4** (3β-O-Acetyl-30-bromobetulinic acid) was obtained as white crystals, 1.1 g (65%): mp 170–172°C. IR (DRIFT): 2600–3400, 1721, 1642 cm^-1^. ^1^H NMR (500 MHz, CDCl_3_): δ 0.84 (s, 3H); 0.85 (s, 3H); 0.86 (s, 3H); 0.94 (s, 3H); 0.99 (s, 3H, H-23, 24, 25, 26, 27); 1.98 (q, 1H, *J* = 8.0 Hz); 2.05 (s, 3H, Ac); 2.20 (dt, 2H, *J*_1_ = 11.5 Hz, *J*_2_ = 2.9 Hz); 2.31 (dd, 1H, *J*_1_ = 12.9 Hz, *J*_2_ = 2.9 Hz); 3.04 (td, 1H, *J*_1_ = 11.2 Hz, *J*_2_ = 4.6 Hz, H-19β); 4.01 (AB-system, 2H, *J*_*GEM*_ = 10.3 Hz, H-30); 4.48 (dd, 1H, *J*_1_ = 10.9 Hz, *J*_2_ = 5.7 Hz, H-3α); 5.05 (s, 1H, H-29 *pro-E*); 5.16 (s, 1H, H-29 *pro-Z*). ^13^C NMR (125 MHz, CDCl_3_): δ = 14.65; 16.08; 16.17; 16.45; 18.14; 20.95; 21.30; 23.67; 26.77; 27.94; 29.68; 32.03; 33.11; 34.25; 36.74; 37.12; 37.14; 37.79; 38.38; 38.41; 40.71; 42.37; 42.99; 50.35; 50.82; 55.40; 56.43; 80.91; 113.46; 151.20; 171.06; 181.89. MS (ESI-): *m*/*z* (%) = 575 (100, [M-H]^-^). HRMS (ESI-) *m*/*z* calcd for C_32_H_49_BrO_4_ [M-H]* 575.2730, found 575.2722 and 577.2710, found 577.2701.

Compound **5** (3β-O-Triphenylsilyl-30-bromobetulinic acid) was obtained as white crystals, 1.8 g (81%): mp 208–210°C. IR (DRIFT): 2600–3400, 1718, 1642 cm^-1^. ^1^H NMR (500 MHz, CDCl_3_): δ 0.84 (s, 3H); 0.88 (s, 3H); 0.92 (s, 3H); 0.94 (s, 3H); 0.96 (s, 3H, H-23, 24, 25, 26, 27); 1.75 (t, 2H, *J* = 10.9 Hz); 1.97 (dd, 1H, *J*_1_ = 12.0 Hz, *J*_2_ = 4.6 Hz); 2.17 (m, 2H); 2.30 (d, 1H, *J* = 9.7 Hz); 3.03 (td, 1H, *J*_1_ = 11.5 Hz, *J*_2_ = 4.6 Hz, H-19β); 3.33 (dd, 1H, *J*_1_ = 12.0 Hz, *J*_2_ = 4.6 Hz, H-3α); 3.99 (s, 2H, H-30); 5.03 (s, 1H, H-29 *pro-E*), 5.14 (s, 1H, H-29 *pro-Z*); 7.37–7.45 (m, 9H); 7.64–7.65 (d, 6H, *J* = 6.3 Hz, 15 × H-Ph). ^13^C NMR (125 MHz, CDCl_3_): δ = 14.68; 16.04; 16.12; 16.32; 18.39; 20.87; 23.67; 26.75; 27.90; 28.45; 29.64; 32.02; 33.04; 34.28; 36.73; 37.00; 38.38; 38.53; 39.56; 40.64; 42.35; 43.04; 50.27; 50.77; 55.22; 56.39; 81.13; 113.44; 127.67; 129.69; 135.36; 135.54; 151.18; 181.78. MS (ESI-): m/z (%) = 791 (100, [M-H]^-^). HRMS (ESI-) m/z calcd for C_48_H_61_BrO_3_Si [M+H]^+^ 791.3495, found 791.3491 and 793.3475, found 793.3480.

#### Azides 6–8

Bromoderivative 4 (2 mmol) was dissolved in DMSO (40 mL) and NaN_3_ (260 mg, 2 equiv.) was added. The reaction mixture was stirred at r.t. for 36 h, the reaction was monitored on TLC in hexane/EtOAc 10: 1. After that, the reaction mixture was worked up in the usual manner and crude product was chromatographed on silica gel (100 g) in gradient hexane/EtOAc 10: 1 to hexane/EtOAc 2: 1.

Compound **6** (3β-*O*-Acetyl-30-azidobetulinic acid) was obtained as 700 mg (65%): mp 148–150°C (hexane). IR (DRIFT): 2600–3400, 2103, 1719, 1642 cm^-1^. ^1^H NMR (500 MHz, CDCl_3_): δ 0.83 (s, 3H); 0.85 (s, 3H); 0.93 (s, 3H); 0.99 (s, 3H); 1.04 (s, 3H, H-23, 24, 25, 26, 27); 2.05 (s, 3H, Ac); 2.29 (d, 1H, *J* = 10.2 Hz); 2.94 (td, 1H, *J*_1_ = 10.9 Hz, *J*_2_ = 4.6 Hz, H-19β); 3.77 (AB-system, 2H, *J*_*GEM*_ = 16.0 Hz, H-30); 4.47 (dd, 1H, *J*_1_ = 10.9 Hz, *J*_2_ = 5.2 Hz, H-3α); 4.99 (s, 1H, H-29 *pro-E*), 5.04 (s, 1H, H-29 *pro-Z*). ^13^C NMR (125 MHz, CDCl_3_): δ = 14.61; 16.03; 16.15; 16.44; 18.11; 20.92; 21.29; 23.64; 26.73; 27.92; 29.65; 31.97; 34.20; 36.72; 37.09; 37.10; 37.77; 38.31; 38.35; 40.68; 42.33; 43.41; 50.29; 55.36; 55.62; 55.63; 56.41; 80.89; 111.55; 148.85; 171.07; 182.21. MS (ESI-): *m*/*z* (%) = 538 (100, [M-H]^-^). HRMS (ESI-TOF) *m*/*z* calcd for C_32_H_49_N_3_O_4_ [M-H]^-^ 538.3639, found 538.3632.

Compound **7** (3β-*O*-Triphenylsilyl-30-azidobetulinic acid) was prepared by the same procedure as compound **6** except CH_3_CN was used as a solvent because of low solubility of **5** in DMSO. The reaction gave 815 mg (54%): mp 171–175°C (hexane). IR (DRIFT): 2600–3400, 2106, 1723, 1490 cm^-1^. ^1^H NMR (500 MHz, CDCl_3_): δ 0.84 (s, 3H); 0.88 (s, 3H); 0.91 (s, 3H); 0.94 (s, 3H); 0.96 (s, 3H, H-23, 24, 25, 26, 27); 1.97 (dd, 1H, *J*_1_ = 12.6 Hz, *J*_2_ = 8.0 Hz); 2.28 (d, 1H, *J* = 9.2 Hz); 2.93 (td, 1H, *J*_1_ = 11.5 Hz, *J*_2_ = 5.2 Hz, H-19β); 3.33 (dd, 1H, *J*_1_ = 12.0 Hz, *J*_2_ = 4.6 Hz, H-3α); 3.75 (AB-system, 2H, *J*_*GEM*_ = 13.8 Hz, H-30); 4.96 (s, 1H, H-29 *pro-E*); 5.02 (s, 1H, H-29 *pro-Z*); 7.36–7.45 (m, 9H); 7.65 (d, 6H, *J* = 8.0 Hz, 15 × H-Ph). ^13^C NMR (125 MHz, CDCl_3_): δ = 14.11; 14.66; 16.00; 16.12; 16.32; 18.38; 20.87; 22.64; 26.72; 27.88; 28.44; 29.63; 31.91; 31.97; 34.26; 36.72; 37.00; 38.29; 38.53; 39.56; 40.63; 42.33; 43.46; 50.27; 55.21; 55.49; 56.37; 81.11; 127.67; 129.69; 135.35; 135.54; 148.84; 181.73. MS (ESI-): *m*/*z* (%) = 754 (100, [M-H]^-^). HRMS (ESI-TOF) *m*/*z* calcd for C_48_H_61_N_3_O_3_Si [M+H]^+^ 754.4398, found 754.4384.

1.5 g (2 mmol) of compound **7** was deprotected according to the general procedure 1 to give colourless crystals of 30-azidobetulinic acid (**8**) 833 mg (84%): mp 171–175°C (CHCl_3_/MeOH). IR (DRIFT): 2600–3400, 2115, 1714, 1640 cm^-1^. ^1^H NMR (500 MHz, CDCl_3_): δ 0.76 (s, 3H); 0.83 (s, 3H); 0.94 (s, 3H); 0.97 (s, 3H); 0.99 (s, 3H, H-23, 24, 25, 26, 27); 1.97 (dd, 1H, *J*_1_ = 12.6 Hz, *J*_2_ = 7.7 Hz); 2.16 (m, 2H); 2.30 (d, 1H, *J* = 12.6 Hz); 2.95 (td, 1H, *J*_1_ = 11.2 Hz, *J*_2_ = 4.8 Hz, H-19β); 3.20 (dd, 1H, *J*_1_ = 11.5 Hz, *J*_2_ = 4.6 Hz, H-3α); 3.78 (AB-system, 2H *J*_*GEM*_ = 14.3 Hz, H-30); 4.98 (s, 1H, H-29 *pro-E*), 5.04 (s, 1H, H-29 *pro-Z*). ^13^C NMR (125 MHz, CDCl_3_): δ = 14.67; 15.32; 16.04; 16.10; 18.24; 20.94; 26.78; 27.33; 27.97; 29.36; 31.94; 34.31; 36.74; 37.19; 38.32; 38.69; 38.85; 40.68; 42.37; 43.46; 50.30; 55.30; 55.54; 56.37; 78.98; 111.50; 113.98; 127.76; 148.88; 181.25. MS (ESI-): *m*/*z* (%) = 496 (100, [M-H]^-^). HRMS (ESI-) *m*/*z* calcd for C_30_H_47_N_3_O_3_ [M-H]^-^ 496.3534, found 496.3525.

#### Acetylated compounds 9a–9h

Compound 9a was obtained from 150 mg (0.28 mmol) 6 by the general procedure using 1 equiv. of alkyne, at 50°C while reaction time was 16 h. The yield of white crystals was 169 mg (95%): mp 177–178°C (hexane). IR (DRIFT): 2600–3400, 1725, 1647 cm^-1^. ^1^H NMR (500 MHz, CDCl_3_): δ 0.83 (s, 3H); 0.85 (s, 6H); 0.92 (s, 3H); 0.97 (s, 3H, H-23, 24, 25, 26, 27); 1.77 (t, 1H, *J* = 11,5 Hz); 1.95 (dd, 1H, *J*_1_ = 8.3 Hz, *J*_2_ = 4.6 Hz); 2.05 (m, 3H, Ac); 2.17 (td, 1H, *J*_1_ = 12.3 Hz, *J*_2_ = 3.4 Hz); 2.29 (m, 1H); 2.99 (td, 1H, *J*_1_ = 10.9 Hz, *J*_2_ = 4.6 Hz, H-19β); 4.48 (dd, 1H, *J*_1_ = 8.2 Hz, *J*_2_ = 6.0 Hz, H-3α); 4.75 (s, 1H, H-29 *pro-E*); 5.00 (AB-system, 2H, *J*_*GEM*_ = 15.5 Hz, H-30); 5.10 (s, 1H, H-29 *pro-Z*); 7.34 (t, 1H, *J* = 7.2 Hz, H-36); 7.43 (t, 2H, *J* = 7.2 Hz, H-35, 37); 7.77 (s, 1H, H-31); 7.84 (d, 2H, *J* = 7.2 Hz, H-34, 38). ^13^C NMR (125 MHz, CDCl_3_): δ = 14.63; 16.02; 16.15; 16.44; 18.10; 20.90; 21.29; 23.64; 26.84; 27.92; 29.60; 29.66; 31.92; 34.18; 36.61; 37.07; 37.76; 38.29; 38.34; 40.66; 42.35; 43.29; 50.27; 50.40; 54.75; 55.34; 56.28; 80.88; 112.03; 120.02; 125.74; 128.17; 128.81; 130.53; 147.97; 149.48; 171.06; 181.39. MS (ESI+): *m*/*z* (%) = 642 (100, [M+H]^+^), 664 (75, [M+Na]^+^). MS (ESI-): *m*/*z* (%) = 640 (100, [M-H]^-^). HRMS (ESI-) *m*/*z* calcd for C_40_H_55_N_3_O_4_ [M-H]^-^ 640.4109, found 640.4098.

Compound **9b** was obtained from 150 mg (0.28 mmol) of **6** by the general procedure using 2 equiv. of alkyne at r.t. while reaction time was 19 h. The yield of white crystals was 160 mg (86%): mp 161–165°C (hexane). IR (DRIFT): 2600–3400, 1720, 1450 cm^-1^. ^1^H NMR (500 MHz, CDCl_3_): δ 0.82 (s, 3H); 0.84 (s, 6H); 0.91 (s, 3H); 0.97 (s, 3H, H-23, 24, 25, 26, 27); 1.77 (t, 1H, *J* = 11.4 Hz); 1.96 (dd, 1H, *J*_1_ = 8.0 Hz, *J*_2_ = 4.4 Hz); 2.05 (s, 3H, Ac); 2.15 (td, 1H, *J*_1_ = 12.2 Hz, *J*_2_ = 3.1 Hz); 2.29 (d, 1H, *J* = 12.5 Hz); 2.99 (td, 1H, *J*_1_ = 10.6 Hz, *J*_2_ = 4.2 Hz, H-19β); 4.47 (dd, 1H, *J*_1_ = 10.4 Hz, *J*_2_ = 4.9 Hz, H-3α); 4.75 (s, 1H, H-29 *pro-E*); 5.05 (t, 2H, *J* = 4.7 Hz, H-30); 5.12 (s, 1H, H-29 *pro-Z*); 7.54 (t, 1H, *J* = 8.0 Hz); 7.66 (t, 1H, *J* = 7.3 Hz); 7.72 (d, 1H, *J* = 7.8 Hz); 7.86 (s, 1H, H-31); 8.03 (d, 1H, *J* = 7.8 Hz, H-35, 36, 37, 38); 10.38 (s, 1H, H-39). ^13^C NMR (125 MHz, CDCl_3_): δ = 14.28; 15.67; 15.81; 16.10; 17.74; 20.54; 20.98; 23.29; 26.56; 27.57; 29.24; 31.55; 33.82; 36.24; 36.72; 37.41; 37.94; 37.99; 40.29; 40.90; 41.99; 42.81; 49.90; 50.07; 53.39; 54.97; 55.99; 56.26; 80.53; 111.95; 123.42; 128.34; 128.49; 129.74; 132.69; 133.44; 144.50; 148.97; 170.78; 181.08; 192.00. MS (ESI+): *m*/*z* (%) = 670 (100, [M+H]^+^), 692 (21, [M+Na]^+^). HRMS (ESI+) *m*/*z* calcd for C_41_H_55_N_3_O_5_ [M+H]^+^ 670.4214, found 670.4211.

Compound **9c** was obtained from 150 mg (0.28 mmol) of **6** by the general procedure using 2 equiv. of alkyne at r.t. while reaction time was 24 h. The yield of white crystals was 131 mg (73%): mp 193–196°C (hexane). IR (DRIFT): 2600–3400, 1721, 1461 cm^-1^. ^1^H NMR (500 MHz, CDCl_3_, referenced to TMS): δ 0.76 (d, 1H, *J* = 10.3 Hz, H-5); 0.80 (s, 3H, H-24); 0.82 (s, 3H, H-26); 0.83 (s, 3H, H-23); 0.90 (s, 3H, H-25); 0.94 (m, 1H, H-1a); 0.95 (s, 3H, H-27); 1.07 (d, 1H, *J* = 12.4 Hz, H-12); 1.18 (dt, 1H, *J*_1_ = 13.4 Hz, *J*_2_ = 2.9 Hz, H-21a); 1.23 (m, 1H, H-2a); 1.24 (m, 1H, H-9); 1.34, (m, 1H, H-6a); 1.36 (m, 2H, H-7); 1.36 (m, 1H, *J* = 13.0 Hz, H-15a); 1.41 (m, 1H, H-2b); 1.42 (m, 1H, 12.4 Hz, H-12b); 1.44 (dd, 1H, *J*_1_ = 12.6 Hz, *J*_2_ = 2.6, H-16a); 1.48 (m, 1H, H-6b); 1.51 (q, 1H, *J* = 13.2 Hz, H-22a); 1.53 (m, 1H, *J* = 12.5 Hz, H-11a); 1.53 (q, 1H, *J* = 13.4 Hz, H-21b); 1.62 (m, 1H, *J* = 12.5 Hz, H-11b); 1.63 (dd, 1H, *J*_1_ = 13.0 Hz, *J*_2_ = 3.5 Hz, H-1b); 1.73 (t, 1H, *J* = 11.4 Hz, H-18); 1.93 (m, 1H, *J* = 13.2 Hz, H-22b); 2.02 (s, 3H, Ac); 2.03 (m, 1H, ∑*J* = 13.0 Hz, H-15b); 2.18 (td, 1H, *J*_1_ = 12.0 Hz, *J*_2_ = 3.0 Hz, H-13); 2.28 (td, 1H, *J*_1_ = 12.6 Hz, *J*_2_ = 2.6, H-16b); 3.00 (td, 1H, *J*_1_ = 11.1 Hz, *J*_2_ = 4.5 Hz, H-19β); 4.45 (m, 1H, *J*_1_ = 11.0 Hz, *J*_2_ = 5.0 Hz, H-3α); 4.69 (s, 1H, H-29 *pro-E*); 4.96 (d, 1H, *J* = 15.6 Hz, H-30a); 5.04 (d, 1H, *J* = 15.6 Hz, H-30b); 5.07 (s, 1H, H-29 *pro-Z*); 7.24 (td, 1H, *J*_1_ = 6.2 Hz, *J*_2_ = 1.0 Hz, H-36); 7.79 (td, 1H, *J*_1_ = 7.8 Hz, *J*_2_ = 1.7 Hz, H-37); 8.20 (dt, 1H, *J*_1_ = 7.9 Hz, *J*_2_ = 1.0 Hz, H-38); 8.20 (s, 1H, H-31); 8.58 (dq, 1H, *J*_1_ = 4.9 Hz, *J*_2_ = 0.8 Hz, H-35). ^13^C NMR (125 MHz, CDCl_3_): δ = 14.73 (C27); 16.14 (C25); 16.25 (C26); 16.55 (C24); 18.22 (C6); 21.01 (C2); 21.39 (Ac, CH_3_); 23.76 (C11); 26.91 (C12); 28.02 (C23); 29.72 (C21); 32.04 (C15); 32.11 (C16); 34.32 (C7); 36.82 (C22); 37.19 (C10); 37.87 (C4); 38.39 (C13); 38.46 (C1); 40.78 (C8); 42.48 (C14); 43.66 (C19); 50.38 (C9); 50.63 (C18); 54.72 (C30); 55.45 (C5); 56.35 (C17); 80.98 (C3); 112.19 (C29); 120.59 (C38); 122.83 (C31); 123.09 (C36); 137.36 (C37); 148.23 (C32); 149.20 (C35); 149.39 (C33); 150.11 (C20); 171.17 (Ac, C = O); 180.72 (C28). MS (ESI+): *m*/*z* (%) = 643 (100, [M+H]^+^), 665 (15, [M+Na]^+^). HRMS (ESI+) *m*/*z* calcd for C_39_H_54_N_4_O_4_ [M+H]^+^ 643.4218, found 643.4220.

Compound **9d** was obtained from 150 mg (0.28 mmol) of **6** by the general procedure using 2 equiv. of alkyne at r.t. while reaction time was 28 h. The yield of white crystals was 135 mg (76%): mp 176–179°C (cyclohexane). IR (DRIFT): 2500–3400, 1723, 1451 cm^-1^. ^1^H NMR (500 MHz, CDCl_3_, referenced to TMS): δ 0.76 (d, 1H, *J* = 10.3 Hz, H-5); 0.80 (s, 3H, H-24); 0.81 (s, 3H, H-25); 0.82 (s, 3H, H-23); 0.90 (s, 3H, H-26); 0.95 (m, 1H, H-1a); 0.96 (s, 3H, H-27); 1.09 (dd, 1H, *J*_1_ = 12.4 Hz, *J*_2_ = 4.6 Hz, H-12a); 1.18 (dt, 1H, *J*_1_ = 13.4 Hz, *J*_2_ = 2.9 Hz, H-21a); 1.24 (m, 1H, H-6a); 1.25 (m, 1H, H-2a); 1.25 (m, 1H, H-9); 1.26 (m, 1H, H-15a); 1.36 (m, 2H, H-7); 1.36 (m, 1H, *J* = 12.4 Hz, H-12b); 1.38 (m, 1H, H-2b); 1.40 (dd, 1H, *J*_1_ = 12.5 Hz, *J*_2_ = 3.6 Hz, H-6b); 1.42 (m, 1H, H-16a); 1.49 (m, 1H, H-22a); 1.53 (m, 1H, H-21b); 1.54 (m, 1H, *J* = 12.5 Hz, H-11a); 1.62 (m, 1H, *J* = 12.5 Hz, H-11b); 1.64 (dd, 1H, *J*_1_ = 13.0 Hz, *J*_2_ = 3.5 Hz, H-1b); 1.75 (t, 1H, *J* = 11.4 Hz, H-18); 1.93 (m, 1H, *J* = 13.2 Hz, H-22b); 1.96 (m, 1H, H-15b); 2.02 (s, 3H, Ac); 2.22 (td, 1H, *J*_1_ = 12.0 Hz, *J*_2_ = 3.0 Hz, H-13); 2.30 (td, 1H, *J*_1_ = 12.6 Hz, *J*_2_ = 2.6 Hz, H-16; 2.97 (td, 1H, *J*_1_ = 11.1 Hz, *J*_2_ = 4.5 Hz, H-19β); 4.45 (dd, 1H, *J*_1_ = 10.5 Hz, *J*_2_ = 5.2 Hz, H-3α); 4.82 (s, 1H, H-29 *pro-E*); 5.00 (d, 1H, *J* = 15.6 Hz, H-30a); 5.04 (d, 1H, *J* = 15.6 Hz, H-29 *pro-Z*); 5.11 (s, 1H, H-29b); 7.39 (m, 1H, *J* = 7.8 Hz, H-37); 7.92 (s, 1H, H-31); 8.28 (dt, 1H, *J*_1_ = 8.0 Hz, *J*_2_ = 1.8 Hz, H-38); 8.55 (dd, 1H, *J*_1_ = 4.9 Hz, *J*_2_ = 1.0 Hz, H-36); 8.98 (d, 1H, *J* = 1.5 Hz, H-34). ^13^C NMR (125 MHz, CDCl_3_): δ = 14.73 (C27); 16.15 (C26); 16.27 (C25); 16.56 (C24); 18.23 (C6); 21.07 (C2); 21.39 (Ac, CH_3_); 23.76 (C11); 27.22 (C12); 28.02 (C23); 29.76 (C21); 32.21 (C16); 32.31 (C15); 34.34 (C7); 36.79 (C22); 37.20 (C10); 37.88 (C4); 38.32 (C13); 38.49 (C1); 40.80 (C8); 42.49 (C14); 43.11 (C19); 50.44 (C9); 50.84 (C18); 55.47 (C5); 55.59 (C30); 56.33 (C17); 80.97 (C3); 112.29 (C29); 120.71 (C31); 124.14 (C37); 127.24 (C33); 133.73 (C38); 144.64 (C32); 146.37 (C34); 148.48 (C36); 149.65 (C20); 171.14 (Ac, C = O); 180.15 (C28). MS (ESI+): *m*/*z* (%) = 643 (100, [M+H]^+^). HRMS (ESI+) *m*/*z* calcd for C_39_H_54_N_4_O_4_ [M+H]^+^ 643.4218, found 643.4221.

Compound **9e** was obtained from 150 mg (0.28 mmol) of **6** by the general procedure using 2 equiv. of alkyne at r.t. while reaction time was 20 h. The yield of white crystals was 144 mg (76%): mp 196–198°C (cyclohexane). IR (DRIFT): 2450–3400, 1731, 1651 cm^-1^. ^1^H NMR (500 MHz, CDCl_3_): δ 0.83 (s, 3H); 0.85 (s, 6H); 0.92 (s, 3H); 0.97 (s, 3H, H-23, 24, 25, 26, 27); 1.76 (t, 1H, *J* = 11,5 Hz); 1.95 (dd, 1H, *J*_1_ = 12.6 Hz, *J*_2_ = 8.0 Hz); 2.05 (m, 3H, Ac); 2.18 (m, 1H); 2.29 (m, 1H); 2.99 (s, 7H, Me_2_N); 2.99 (m, 1H, H-19β); 4.48 (dd, 1H, *J*_1_ = 8.2 Hz, *J*_2_ = 6.0 Hz, H-3α); 4.74 (s, 1H, H-29 *pro-E*); 4.98 (AB-system, 2H, *J*_*GEM*_ = 15.5 Hz, H-30); 5.08 (s, 1H, H-29 *pro-Z*); 6.77 (s, 1H, aniline); 6.79 (s, 1H, aniline); 7.63 (s, 1H, H-31); 7.70 (s, 1H, aniline); 7.71 (s, 1H, aniline). ^13^C NMR (125 MHz, CDCl_3_): δ = 14.62; 16.03; 16.15; 16.45; 18.11; 20.91; 21.29; 23.66; 26.80; 27.93; 29.61; 31.17; 31.87; 31.93; 34.18; 36.62; 37.09; 37.77; 38.27; 38.35; 40.53; 40.66; 42.36; 43.36; 50.28; 50.34; 54.58; 55.34; 56.26; 80.89; 111.87; 112.58; 118.58; 121.52; 126.69; 148.40; 149.60; 150.37; 171.02; 180.99. MS (ESI+): *m*/*z* (%) = 485 (100, [M+H]^+^). HRMS (ESI+) *m*/*z* calcd for C_42_H_60_N_4_O_4_ [M+H]^+^ 685.4687, found 685.4687.

Compound **9f** was obtained from 150 mg (0.28 mmol) of **6** by the general procedure using 1 equiv. of alkyne at r.t. while reaction time was 20 h. The yield of white crystals was 165 mg (85%): mp 193–196°C (hexane). IR (DRIFT): 2600–3500, 1724, 1655 cm^-1^. ^1^H NMR (500 MHz, CDCl_3_): δ 0.83 (s, 3H); 0.85 (s, 6H); 0.92 (s, 3H); 0.98 (s, 3H, H-23, 24, 25, 26, 27); 1.35 (s, 9H, H-t-Bu); 1.78 (t, 1H, *J* = 11.4 Hz); 2.05 (s, 3H, Ac); 2.17 (td, 1H, *J*_1_ = 12.5 Hz, *J*_2_ = 2.6 Hz); 2.30 (dt, 1H, *J*_1_ = 13.0 Hz, *J*_2_ = 3.1 Hz); 2.99 (td, 1H, *J*_1_ = 10.9 Hz, *J*_2_ 4.2 Hz, H-19β); 4.48 (dd, 1H, *J*_1_ = 10.4 Hz, *J*_2_ = 4.7 Hz, H-3α); 4.74 (s, 1H, H-29 *pro-E*); 5.00 (AB-system, 2H, *J*_*GEM*_ = 16.6 Hz, H-30); 5.09 (s, 1H, H-29 *pro-Z*); 7.46 (d, 2H, *J* = 8.8 Hz, H-35, 37); 7.74 (s, 1H, H-31); 7.78 (d, 2H, J = 8.3 Hz, H-34, 38). ^13^C NMR (125 MHz, CDCl_3_): δ = 14.62; 16.02; 16.14; 16.44; 18.10; 20.90; 21.31; 23.65; 26.83; 27.91; 29.60; 29.68; 31.26; 31.92; 34.16; 34.64; 36.59; 37.08; 37.76; 38.27; 38.34; 40.64; 42.34; 43.22; 50.25; 50.36; 54.76; 55.33; 56.27; 80.87; 111.86; 119.74; 125.47; 125.72; 127.70; 147.93; 149.53; 151.29; 171.06; 181.43. MS (ESI-): *m*/*z* (%) = 696 (100, [M-H]^-^). HRMS (ESI-) *m*/*z* calcd for C_44_H_63_N_3_O_4_ [M-H]^-^ 696.4735, found 696.4723.

Compound **9g** was obtained from 150 mg (0.28 mmol) of **6** by the general procedure using 1 equiv. of alkyne at 50°C while reaction time was 18 h. The yield of white crystals was 147 mg (83%): mp 158–159°C (hexane). IR (DRIFT): 2600–3400, 1726, 1661 cm^-1^. ^1^H NMR (500 MHz, CDCl_3_): δ 0.83 (s, 3H); 0.85 (s, 6H); 0.91 (s, 3H); 0.94 (s, 3H, H-23, 24, 25, 26, 27); 1.67 (m, 8H, H-34, 35, 36, 37) 2.05 (s, 3H, Ac); 2.29 (d, 1H, *J* = 12.9 Hz); 2.95 (td, 1H, *J*_1_ = 10.9 Hz, *J*_2_ = 4.3 Hz, H-19β); 3.21 (t, 1H, *J* = 8.3 Hz, H-33); 4.48 (dd, 1H, *J*_1_ = 10.9 Hz, *J*_2_ = 5.2 Hz, H-3α); 4.69 (s, 1H, H-29 *pro-E*); 4.91 (AB-system, 2H, *J*_*GEM*_ = 15.8 Hz, H-30); 5.05 (s, 1H, H-29 *pro-Z*); 7.26 (s, 1H, H-31). ^13^C NMR (125 MHz, CDCl_3_): δ = 14.59; 14.62; 15.99; 16.03; 16.12; 16.16; 16.46; 18.10; 20.90; 21.28; 23.65; 25.11; 26.68; 27.93; 29.59; 29.66; 31.90; 33.26; 34.18; 36.61; 36.68; 37.07; 37.76; 38.24; 28.36; 40.64; 42.34; 43.26; 50.20; 54.47; 55.34; 56.27; 80.87; 111.89; 120.05; 149.61; 152.93; 171.04; 180.94. MS (ESI-): *m*/*z* (%) = 632 (100, [M-H]^-^). HRMS (ESI-) *m*/*z* calcd for C_39_H_59_N_3_O_4_ [M-H]^-^ 632.4422, found 632.4414.

Compound **9h** was obtained from 300 mg (0.56 mmol) of **6** by the general procedure using 2 equiv. of alkyne at r.t. while reaction time was 30 h. The yield of white crystals was 33 mg (10%): mp 178–184°C (cyclohexane). IR (DRIFT): 3650, 2600–3500, 1726, 1643 cm^-1^. ^1^H NMR (500 MHz, CDCl_3_): δ 0.84 (s, 3H); 0.85 (s, 6H); 0.93 (s, 3H); 0.97 (s, 3H, H-23, 24, 25, 26, 27); 2.05 (s, 3H, Ac); 2.30 (bd, 1H, *J* = 12.9 Hz); 2.95 (m, 1H, H-19β); 4.48 (dd, 1H, *J*_1_ = 10.9 Hz, *J*_2_ = 5.2 Hz, H-3α); 4.70 (s, 1H, H-29 *pro-E*); 4.85–5.20 (m, 2H, C**H**_**2**_-NH_2_); 5.03 (AB-system, 2H, *J*_*GEM*_ = 15.8 Hz, H-30); 5.13 (s, 1H, H-29 *pro-Z*); 8.13 (s, 1H, H-31); 10.17 (s, NH_2_). ^13^C NMR (125 MHz, CDCl_3_): δ = 14.61; 16.01; 16.17; 16.46; 18.12; 20.90; 21.29; 23.65; 27.05; 27.93; 29.58; 31.97; 34.22; 36.20; 37.10; 37.79; 38.23; 38.39; 40.69; 42.37; 43.11; 50.29; 50.84; 55.34; 55.38; 56.33; 64.17; 77.20; 80.82; 112.57; 125.64; 132.51; 148.84; 171.02; 185.11. MS (ESI+): *m*/*z* (%) = 595 (100, [M+H]^+^). HRMS (ESI+) *m*/*z* calcd for C_35_H_54_N_4_O_4_ [M+H]^+^ 595.4218, found 595.4220.

#### Silylated compounds 10a–10g

Compound 10a was obtained from 150 mg (0.20 mmol) of 7 by the general procedure using 1 equiv. of alkyne at 50°C while reaction time was 20 h. The yield of white crystals was 157 mg (92%): mp 164–166°C (cyclohexane). IR (DRIFT): 2600–3400, 1734, 1652 cm^-1^. ^1^H NMR (500 MHz, CDCl_3_): δ 0.83 (s, 3H); 0.89 (s, 3H); 0.90 (s, 3H); 0.92 (s, 3H); 0.96 (s, 3H, H-23, 24, 25, 26, 27); 2.15 (dd, 1H, *J*_1_ = 12.5 Hz, *J*_2_ = 3.1 Hz); 2.29 (d, 1H, *J* = 12.5 Hz); 3.00 (td, *J*_1_ = 10.9 Hz, *J*_2_ = 4.4 Hz, 1H, H-19β); 3.34 (dd, 1H, *J*_1_ = 11.9 Hz, *J*_2_ = 4.2 Hz, H-3α); 4.70 (s, 1H, H-29 *pro-E*); 4.99 (AB-system, 2H, *J*_*GEM*_ = 15.6 Hz, H-30); 5.07 (s, 1H, H-29 *pro-Z*); 7.31–7.46 (m, 12H, H-35, 36, 37, 3 × Ph); 7.66 (dd, 6H, *J*_1_ = 7.8 Hz, *J*_2_ = 1.3 Hz, 3 × Ph); 7.75 (s, 1H, H-31); 7.84 (d, 2H, *J* = 8.3 Hz, H-34, 38). ^13^C NMR (125 MHz, CDCl_3_): δ = 14.66; 15.98; 16.10; 16.32; 18.35; 20.82; 26.81; 27.88; 28.42; 29.56; 31.81; 31.90; 34.21; 36.59; 36.95; 38.27; 28.52; 39.54; 40.59; 42.33; 43.38; 50.20; 50.31; 54.55; 55.16; 56.25; 81.08; 111.91; 120.01; 125.73; 127.67; 128.15; 128.80; 129.70; 130.53; 135.32; 135.53; 147.95; 149.43; 181.32. MS (ESI-): *m*/*z* (%) = 856 (100, [M-H]^-^). HRMS (ESI-) *m*/*z* calcd for C_56_H_67_N_3_O_3_Si [M-H]^-^ 856.4868, found 856.4849.

Compound **10b** was obtained from 150 mg (0.20 mmol) of **7** by the general procedure using 2 equiv. of alkyne at r.t. while reaction time was 22 h. The yield of white crystals was 148 mg (84%): mp 161–163°C (cyclohexane). IR (DRIFT): 2600–3400, 1724, 1642 cm^-1^. ^1^H NMR (500 MHz, CDCl_3_): δ 0.83 (s, 3H); 0.88 (s, 3H); 0.90 (s, 3H); 0.92 (s, 3H); 0.96 (s, 3H, H-23, 24, 25, 26, 27); 2.14 (td, 1H, *J*_1_ = 12.6 Hz, *J*_2_ = 2.6 Hz); 2.29 (d, 1H, *J* = 12.6 Hz); 2.99 (td, *J*_1_ = 10.9 Hz, *J*_2_ = 4.3 Hz, 1H, H-19β); 3.34 (dd, 1H, *J*_1_ = 11.7 Hz, *J*_2_ = 4.3 Hz, H-3α); 4.75 (s, 1H, H-29 *pro-E*); 5.04 (AB-system, 2H, *J*_*GEM*_ = 15.8 Hz, H-30); 5.10 (s, 1H, H-29 *pro-Z*); 7.34–7.45 (m, 9H, 3 × Ph); 7.51 (t, 1H, *J* = 7.7 Hz, H-36); 7.62 (m, 1H, H-37); 7.66 (m, 6H, 3 × Ph); 7.72 (d, 1H, J = 8.6 Hz, H-38); 7.83 (s, 1H, H-31); 8.03 (d, 2H, J = 8.9 Hz, H-35); 10.38 (s, 1H, H-39). ^13^C NMR (125 MHz, CDCl_3_): δ = 14.67; 15.98; 16.11; 16.32; 18.36; 20.84; 22.63; 26.87; 27.88; 28.43; 29.56; 31.56; 31.89; 34.23; 36.58; 38.29; 38.54; 39.54; 40.60; 42.35; 43.28; 50.22; 54.37; 54.78; 55.18; 56.27; 81.08; 112.24; 119.89; 123.71; 127.67; 128.63; 128.83; 129.70; 130.07; 133.05; 133.72; 133.84; 135.34; 135.53; 144.83; 149.30; 192.27. MS (ESI+): *m*/*z* (%) = 886 (100, [M+H]^+^), 908 (8, [M+Na]^+^). HRMS (ESI+) *m*/*z* calcd for C_57_H_67_N_3_O_4_Si [M+H]^+^ 886.4974, found 886.4975.

Compound **10c** was obtained from 150 mg (0.20 mmol) of **7** by the general procedure using 2 equiv. of alkyne at r.t. while reaction time was 26 h. The yield of white crystals was 130 mg (75%): mp 197–200°C (cyclohexane). IR (DRIFT): 2600–3400, 1718, 1641 cm^-1^. ^1^H NMR (500 MHz, CDCl_3_): δ 0.82 (s, 3H); 0.88 (s, 3H); 0.89 (s, 3H); 0.92 (s, 3H); 0.95 (s, 3H, H-23, 24, 25, 26, 27); 2.17 (t, 1H, *J* = 11.7 Hz); 2.28 (d, 1H, *J* = 11.7 Hz); 3.01 (td, 1H, *J*_1_ = 10.4 Hz, *J*_2_ = 6.5 Hz, H-19β); 3.33 (dd, 1H, *J*_1_ = 12.2 Hz, *J*_2_ = 3.6 Hz, H-3α); 4.66 (s, 1H, H- 29 *pro-E*); 5.01 (AB-system, 2H, *J*_*GEM*_ = 15.8 Hz, H-30); 5.06 (s, 1H, H-29 *pro-Z*); 7.24 (m, 1H, H-36); 7.36–7.45 (m, 9H, 3 × Ph); 7.65 (d, 6H, *J* = 6.5 Hz, 3 × Ph); 7.79 (t, 1H, *J* = 7.8 Hz, H-35); 8.19 (s, 1H, H-31); 8.21 (d, 1H, *J* = 8.8 Hz, H-37); 8.59 (d, 1H, *J* = 4.7 Hz, H-34). ^13^C NMR (125 MHz, CDCl_3_): δ = 14.65; 15.99; 16.10; 16.33; 18.36; 20.81; 26.79; 27.87; 28.42; 29.68; 31.56; 32.00; 34.23; 36.70; 36.95; 38.24; 38.49; 39.53; 40.59; 42.33; 43.54; 50.19; 50.43; 54.52; 55.16; 56.21; 81.09; 111.83; 119.88; 122.69; 127.50; 127.67; 129.70; 135.32; 136.30; 137.21; 142.18; 148.13; 149.09; 149.98, 181.13. MS (ESI+): *m*/*z* (%) = 859 (100, [M+H]^+^), 881 (12, [M+Na]^+^). HRMS (ESI+) *m*/*z* calcd for C_55_H_66_N_4_O_3_Si [M+H]^+^ 859.4977, found 859.4977.

Compound **10d** was obtained from 150 mg (0.20 mmol) of **7** by the general procedure using 2 equiv. of alkyne at r.t. while reaction time was 36 h. The yield of white crystals was 131 mg (77%): mp 202–204°C (cyclohexane). IR (DRIFT): 2650–3450, 1729, 1646 cm^-1^. ^1^H NMR (500 MHz, CDCl_3_): δ 0.82 (s, 3H); 0.88 (s, 3H); 0.89 (s, 3H); 0.91 (s, 3H); 0.93 (s, 3H); 0.95 (s, 3H, H-23, 24, 25, 26, 27); 2.30 (d, 1H, *J* = 12.6 Hz); 2.98 (td, 1H, *J*_1_ = 10.6 Hz, *J*_2_ = 4.0 Hz, H-19β); 3.33 (dd, 1H, *J*_1_ = 11.7 Hz, *J*_2_ = 4.0 Hz, H-3α); 4.79 (s, 1H, H-29 *pro-E*); 5.01 (AB-system, 2H, *J*_*GEM*_ = 16.6 Hz, H-30); 5.10 (s, 1H, H-29 *pro-Z*); 7.36–7.45 (m, 9H, 3 × Ph); 7.65 (d, 6H, 3 × Ph); 7.89 (s, 1H, H-31); 8.28 (d, 1H, *J* = 7.7 Hz, H-37); 8.57 (d, 1H, *J* = 3.7 Hz, H-35); 8.99 (s, 1H, H-34). ^13^C NMR (125 MHz, CDCl_3_): δ = 14.67; 16.01; 16.13; 16.34; 18.39; 20.91; 27.11; 27.89; 28.44; 29.62; 32.07; 32.13; 34.27; 36.66; 36.99; 38.21; 28.56; 39.55; 40.65; 42.38; 43.02; 50.27; 50.72; 55.20; 55.40; 56.19; 81.10; 112.12; 120.54; 123.97; 127.07; 127.68; 129.70; 133.51; 135.35; 135.54; 144.57; 146.40; 148.50; 149.52; 181.13. MS (ESI+): *m*/*z* (%) = 859 (100, [M+H]^+^), 881 (7, [M+Na]^+^). HRMS (ESI+) *m*/*z* calcd for C_55_H_66_N_4_O_3_Si [M+H]^+^ 859.4977, found 859.4974.

Compound **10e** was obtained from 150 mg (0.20 mmol) of **7** by the general procedure using 2 equiv. of alkyne at r.t. while reaction time was 24 h. The yield of white crystals was 128 mg (72%): mp 194–196°C (cyclohexane). IR (DRIFT): 2600–3400, 1724, 1652 cm^-1^. ^1^H NMR (500 MHz, CDCl_3_, referenced to TMS): δ 0.53 (d, 1H, *J* = 10.3 Hz, H-5); 0.64 (td, 1H, *J*_1_ = 13.2 Hz, *J*_2_ = 3.5 Hz, H-1a); 0.82 (s, 3H, H-25); 0.86 (s, 3H, H-24); 0.88 (s, 3H, H-27); 0.92 (s, 3H, H-26); 0.94 (s, 3H, H-23); 1.02 (qd, 1H, *J*_1_ = 12.4 Hz, *J*_2_ = 3.8 Hz, H-12a); 1.16 (t, 1H, *J* = 12.5 Hz, H-9); 1.19 (dd, 1H, *J*_1_ = 13.4 Hz, *J*_2_ = 2.9 Hz, H-21a); 1.28 (m, 1H, *J* = 12.5 Hz, H-2a); 1.31 (m, 2H, H-7); 1.33 (m, 1H, H-6a); 1.38 (m, 1H, *J* = 12.4 Hz, H-12b); 1.41 (dd, 1H, *J*_1_ = 12.5 Hz, *J*_2_ = 2.2 Hz, H-2b); 1.44 (m, 1H, H-15a); 1.45 (m, 1H, *J* = 12.6 Hz, H-16a); 1.49 (m, 1H, *J* = 13.5 Hz, H-22a); 1.50 (mm, 4H, H-11a, H-1, 6, 21b); 1.74 (t, 1H, *J* = 11.4 Hz, H-18); 1.74 (m, 1H, H-11b); 1.92 (m, 1H, *J* = 13.5 Hz, H-22b); 1.99 (m, 1H, *J* = 13.0 Hz, H-15b); 2.13 (td, 1H, *J*_1_ = 12.0 Hz, *J*_2_ = 3.0 Hz, H-13); 2.26 (dt, 1H, *J*_1_ = 12.6 Hz, *J*_2_ = 2.6 Hz, H-16b); 2.95 (td, 1H, *J*_1_ = 11.1 Hz, *J*_2_ = 4.5 Hz, H-19β); 2.98 (s, 6H, H-40, 41); 3.32 (dd, 1H, *J*_1_ = 11.0 Hz, *J*_2_ = 5.0 Hz, H-3α); 4.67 (s, 1H, H-29 *pro-E*); 4.94 (m, 2H, H-30); 5.03 (s, 1H, H-29 *pro-Z*); 6.80 (br, 2H, H-35, 37); 7.36 (t, 6H, *J* = 7.2 Hz, H-3´,3´´a,b,c); 7.42 (tt, 3H, *J*_1_ = 7.2 Hz, *J*_2_ = 1.5 Hz, H-4´a,b,c); 7.60 (s, 1H, H-31); 7.63 (d, 6H, *J* = 7.2 Hz, H-2´,2´´a,b,c); 7.69 (d, 2H, *J* = 8.2 Hz, H-34,38). ^13^C NMR (125 MHz, CDCl_3_): δ = 14.79 (C27); 16.11 (C26); 16.22 (C25); 16.44 (C24); 18.46 (C6); 20.97 (C2); 26.92 (C12); 28.00 (C11); 28.54 (C23); 29.70 (C21); 31.96 (C15); 32.05 (C16); 34.36 (C7); 36.71 (C22); 37.10 (C10); 38.38 (C13); 38.66 (C1); 39.67 (C4); 40.74 (C8); 40.86 (C40,C41); 42.47 (C14); 43.41 (C19); 50.34 (C18); 50.43 (C9); 54.65 (C29); 56.30 (C5); 56.32 (C17); 91.22 (C3); 111.62 (C29); 112.95 (C35, 37); 118.76 (C31); 122.23 (C33); 126.84 (C34,C38); 127.77 (Ca,b,c3´,3´´); 129.81 (Ca,b,c4´); 135.46 (Ca,b,c1´,1´´); 135.64 (Ca,b,c2´,2´´); 148.42 (C32); 149.70 (C20); 150.21 (C36); 180.72 (C28). MS (ESI+): *m*/*z* (%) = 901 (100, [M+H]^+^), 923 (6, [M+Na]^+^). HRMS (ESI+) *m*/*z* calcd for C_58_H_72_N_4_O_3_Si [M+H]^+^ 901.5446, found 901.5441.

Compound **10f** was obtained from 150 mg (0.20 mmol) of **7** by the general procedure using 1 equiv. of alkyne at r.t. while reaction time was 24 h. The yield of white crystals was 147 mg (81%): mp 195–197°C (cyclohexane). IR (DRIFT): 2600–3400, 1717, 1639 cm^-1^. ^1^H NMR (500 MHz, CDCl_3_): δ 0.84 (s, 3H); 0.89 (s, 3H); 0.90 (s, 3H); 0.95 (s, 3H); 0.96 (s, 3H, H-23, 24, 25, 26, 27); 1.35 (s, 9H, H-40, 41, 42); 1.76 (t, 2H, *J* = 1.2 Hz); 2.15 (td, 1H, *J*_1_ = 12.6 Hz, *J*_2_ = 2.9 Hz); 2.29 (d, 1H, *J* = 12.9 Hz); 2.98 (td, 1H, *J*_1_ = 11.2 Hz, *J*_2_ = 4.9 Hz, H-19β); 3.35 (dd, 1H, *J*_1_ = 11.7 Hz, *J*_2_ = 4.3 Hz, H-3α); 4.69 (s, 1H, H-29 *pro-E*); 4.98 (s, 2H, H-30); 5.05 (s, 1H, H-29 *pro-Z*); 7.37–7.47 (m, 11H, H-35, 37, 3 × Ph); 7.66 (d, 6H, *J* = 6.6 Hz, 3 × Ph); 7.78 (d, 2H, H-34, 38). ^13^C NMR (125 MHz, CDCl_3_): δ = 14.68; 16.00; 16.11; 16.33; 18.36; 20.84; 26.82; 27.89; 28.43; 29.58; 29.67; 31.26; 31.93; 34.22; 34.63; 36.59; 36.98; 38.26; 38.52; 39.55; 40.60; 42.34; 43.22; 50.21; 50.34; 54.37; 54.67; 55.18; 56.24; 81.09; 111.63; 119.73; 125.46; 125.71; 127.67; 129.70; 135.34; 135.52; 147.91; 149.58; 151.27; 181.28. MS (ESI+): *m*/*z* (%) = 914 (100, [M+H]^+^), 937 (75, [M+Na]^+^). HRMS (ESI+) *m*/*z* calcd for C_60_H_75_N_3_O_3_Si [M+H]^+^ 914.5650, found 914.5650.

Compound **10g** was obtained from 150 mg (0.20 mmol) of **7** by the general procedure using 1 equiv. of alkyne at 50°C while reaction time was 15 h. The yield of white crystals was 148 mg (88%): mp 146–148°C (cyclohexane). IR (DRIFT): 2650–3400, 1730, 1452 cm^-1^. ^1^H NMR (500 MHz, CDCl_3_): δ 0.84 (s, 3H); 0.89 (s, 9H); 0.96 (s, 3H, H-23, 24, 25, 26, 27); 2.29 (d, 1H, J = 11.5 Hz); 2.97 (t, 1H, *J* = 12.9 Hz, H-33); 3.21 (td, 1H, *J*_1_ = 15.5 Hz, *J*_2_ = 7.7 Hz, H-19β); 3.34 (d, 1H, *J* = 10.9 Hz, H-3α); 4.65 (s, 1H, H-29 *pro-E*); 4.89 AB-system, 2H, *J*_*GEM*_ = 15.8 Hz, H-30); 5.03 (s, 1H, H-29 *pro-Z*); 7.25 (s, 1H, H-31); 7.37–7.45 (m, 9H, 3 × Ph); 7.66 (d, 6H, *J* = 8.0 Hz, 3 × Ph). ^13^C NMR (125 MHz, CDCl_3_): δ = 14.65; 15.99; 16.10; 16.32; 18.37; 20.82; 25.11; 26.66; 27.89; 28.42; 29.58; 29.66; 31.76; 31.93; 33.23; 34.23; 36.69; 36.97; 38.20; 38.53; 39.53; 40.58; 42.32; 43.47; 50.11; 50.22; 54.19; 55.20; 56.29; 81.11; 111.92; 120.00; 127.65; 129.68; 135.32; 135.50; 149.62; 152.89; 181.13. MS (ESI+): *m*/*z* (%) = 850 (100, [M+H]^+^). HRMS (ESI+) *m*/*z* calcd for C_55_H_71_N_3_O_3_Si [M+H]^+^ 850.5337, found 850.5334.

#### Unprotected compounds 11a–11g

Compound 11a was obtained from 150 mg (0.17 mmol) of 10a by the general deprotection procedure 1 at 60°C for 18 h. The yield of white crystals was 143 mg (79%): mp 137–138°C (CH_2_Cl_2_). IR (DRIFT): 2600–3400, 1724, 1650 cm^-1^. ^1^H NMR (500 MHz, CDCl_3_): δ 0.76 (s, 3H); 0.82 (s, 3H); 0.92 (s, 3H); 0.98 (s, 6H, H-23, 24, 25, 26, 27); 2.18 (dd, 1H, *J*_1_ = 13.5 Hz, *J*_2_ = 3.4 Hz); 2.30 (d, 1H, *J* = 12.7 Hz); 3.00 (td, 1H, *J*_1_ = 11.2 Hz, *J*_2_ = 4.6 Hz, H-19β); 3.21 (dd, 1H, *J*_1_ = 11.2 Hz, *J*_2_ = 4.9 Hz, H-3α); 4.72 (s, 1H, H-29 *pro-E*); 5.00 (AB-system, 2H, *J*_*GEM*_ = 15.8 Hz, H-30); 5.10 (s, 1H, H-29 *pro-Z*); 7.34 (tt, 1H, *J*_1_ = 7.3 Hz, *J*_2_ = 1.3 Hz, H-36); 7.43 (t, 2H, *J* = 7.3 Hz, H-35, 37); 7.77 (s, 1H, H-31); 7.84 (d, 2H, *J* = 7.0 Hz, H-34, 38). ^13^C NMR (125 MHz, CDCl_3_): δ = 14.66; 15.32; 16.01; 16.08; 18.21; 20.89; 26.84; 27.29; 27.94; 29.60; 29.67; 31.86; 31.92; 34.25; 36.62; 37.14; 38.30; 38.66; 38.81; 40.63; 42.36; 43.39; 50.36; 54.59; 55.24; 56.25; 78.95; 111.95; 120.02; 125.74; 128.16; 128.81; 130.54; 147.97; 149.48; 180.97. MS (ESI+): *m*/*z* (%) = 600 (100, [M+H]^+^). HRMS (ESI+) *m*/*z* calcd for C_38_H_53_N_3_O_3_ [M+H]^+^ 600.4160, found 600.4162. Note: deprotection procedure 2 at r.t. for 4 h was also tried with the yield of 76%.

Compound **11b** was obtained from 150 mg (0.17 mmol) of **10b** by the general deprotection procedure 1 at r.t. for 32 h. The yield of white crystals was 117 mg (62%): mp 127–129°C (CH_2_Cl_2_). IR (DRIFT): 2600–3400, 1727, 1453 cm^-1^. ^1^H NMR (500 MHz, CDCl_3_, referenced to TMS): δ 0.66 (d, 1H, *J* = 9.0 Hz, H-5); 0.73 (s, 3H, H-25); 0.80 (s, 3H, H-24); 0.88 (m, 1H, *J* = 13.2 Hz, H-1a); 0.90 (s, 3H, H-26); 0.95 (s, 3H, H-23); 0.96, (s, 3H, H-27); 1.04 (dd, 1H, *J*_1_ = 11.7 Hz, *J*_2_ = 4.2 Hz, H-12a); 1.23 (m, 1H, H-9); 1.24 (mm, 2H, H-15, 21a); 1.25 (m, 1H, H-11a); 1.36 (mm, 2H, H-7); 1.36 (m, 1H, H-6a); 1.38 (m, 1H, H-12b); 1.44 (m, 1H, H-11b); 1.44 (m, 1H, *J* = 12.2 Hz, H-16a); 1.50 (m, 1H, H-15b); 1.52 (mm, 3H, H-2,6,21b); 1.52 (m, 1H, *J* = 13.1 Hz, H-22a); 1.61 (m, 1H, H-2b); 1.63 (m, 1H, *J* = 13.2 Hz, H-1b); 1.75 (t, 1H, *J* = 11.4 Hz, H-18); 1.95 (m, 1H, H-22b); 2.16 (td, 1H, *J*_1_ = 12.1 Hz, *J*_2_ = 3.3 Hz, H-13); 2.28 (dt, 1H, *J*_1_ = 12.2 Hz, *J*_2_ = 3.1 Hz, H-16b); 2.98 (td, 1H, *J*_1_ = 11.1 Hz, *J*_2_ = 4.3 Hz, H-19β); 3.18 (dd, 1H, *J*_1_ = 11.3 Hz, *J*_2_ = 4.7 Hz, H-3α); 4.75 (s, 1H, H-29); 5.00 (d, 1H, *J* = 15.6 Hz, H-30); 5.06 (d, 1H, *J* = 15.6 Hz, H-30b); 5.10 (s, 1H, H-29); 7.51 (tt, 1H, *J*_1_ = 7.5 Hz, *J*_2_ = 0.8 Hz, H-36); 7.64 (td, 1H, *J*_1_ = 7.5 Hz, *J*_2_ = 1.5 Hz, H-37); 7.71 (dd, 1H, *J*_1_ = 7.8 Hz, *J*_2_ = 1.1 Hz, H-38); 7.82 (s, 1H, H-31); 8.02 (dd, 1H, *J*_1_ = 7.8, *J*_2_ = 1.2 Hz, H-35); 10.36 (d, 1H, *J* = 0.5 Hz, H-39). ^13^C NMR (125 MHz, CDCl_3_): δ = 14.78 (C27); 15.43 (C25); 16.12 (C26); 16.20 (C24); 18.34 (C6); 21.02 (C11); 27.06 (C12); 27.44 (C2); 28.06 (C23); 29.71 (C21); 29.77 (C15); 32.02 (C16); 34.38 (C7); 36.71 (C22); 37.28 (C10); 38.41 (C13); 38.80 (C1); 38.94 (C4); 40.77 (C8); 42.49 (C14); 43.39 (C19); 50.50 (C18); 50.52 (C9); 54.93 (C30); 55.38 (C5); 56.35 (C17); 79.03 (C3); 112.37 (C29); 123.84 (C31); 128.76 (C36); 128.96 (C35); 130.20 (C38); 133.18 (C33); 133.83 (C37); 133.96 (C34); 144.93 (C32); 149.45 (C20); 180.36 (C28); 192.41 (C39). MS (ESI+): *m*/*z* (%) = 628 (100, [M+H]^+^). HRMS (ESI-TOF) *m*/*z* calcd for C_39_H_53_N_3_O_4_ [M+H]^+^ 628.4109, found 628.4111.

Compound **11c** was obtained from 150 mg (0.30 mmol) of **10c** by the general deprotection procedure 1 at r.t. for 28 h. The yield of white crystals was 105 mg (58%): mp 141–142°C (CH_2_Cl_2_). IR (DRIFT): 2500–3450, 1735, 1654 cm^-1^. ^1^H NMR (500 MHz, CDCl_3_): δ 0.76 (s, 3H); 0.82 (s, 3H); 0.92 (s, 3H); 0.97 (s, 3H); 0.98 (s, 3H, H-23, 24, 25, 26, 27); 1.75 (t, 1H, *J* = 11.5 Hz); 1.97 (dd, 1H, *J*_1_ = 12.6 Hz, *J*_2_ = 8.3 Hz); 2.06 (m, 1H); 2.22 (td, 1H, *J*_1_ = 13.9 Hz, *J*_2_ = 3.5 Hz); 2.31 (dt, 1H, *J*_1_ = 12.5 Hz, *J*_2_ = 3.2 Hz); 3.04 (td, 1H, *J*_1_ = 11.2 Hz, *J*_2_ = 4.9 Hz, H-19β); 3.21 (dd, 1H, *J*_1_ = 11.6 Hz, *J*_2_ = 4.9 Hz, H-3α); 4.67 (s, 1H, H-29 *pro-E*); 5.04 (AB-system, 2H, *J*_*GEM*_ = 15.7 Hz, H-30); 5.09 (s, 1H, H-29 *pro-Z*); 7.26 (m, 1H, H-pyridine); 7.81 (td, 1H, *J*_1_ = 7.7 Hz, *J*_2_ = 1.7 Hz, H-pyridine); 8.22 (s, 1H, H-31); 8.22 (m, 1H, H-pyridine); 8.61 (dq, *J*_1_ = 4.9 Hz, *J*_2_ = 0.8 Hz, H-pyridine). ^13^C NMR (125 MHz, CDCl_3_): δ = 14.66; 15.34; 16.10; 18.23; 20.90; 26.81; 27.31; 27.96; 29.62; 31.17; 31.95; 32.02; 34.29; 36.72; 37.16; 38.28; 38.72; 38.82; 40.66; 42.38; 43.59; 50.39; 50.46; 54.47; 55.27; 56.21; 78.93; 111.81; 120.49; 122.76; 122.97; 137.24; 148.11; 149.07; 149.35; 150.01; 180.18. MS (ESI-): *m*/*z* (%) = 599 (100, [M-H]^-^). HRMS (ESI-) *m*/*z* calcd for C_37_H_52_N_4_O_3_ [M-H]^-^ 599.3956, found 599.3947. Note: deprotection procedure 2 was also tried with yield of 58% and the attempt to prepare compound **11c** from free azide **8** by the general procedure for click reaction gave the best yield of 74%.

Compound **11d** was obtained from 150 mg (0.30 mmol) of **10d** by the general deprotection procedure 1 at r.t. for 28 h. The yield of white crystals was 112 mg (62%): mp 141–142°C (CH_2_Cl_2_). IR (DRIFT): 2600–3400, 1734, 1645 cm^-1^. ^1^H NMR (500 MHz, DMSO): δ 0.64 (s, 3H); 0.75 (s, 3H); 0.85 (s, 3H); 0.87 (s, 3H); 0.93 (s, 3H, H-23, 24, 25, 26, 27); 2.12 (m, 2H) 2.18 (m, 1H); 2.97 (td, 1H, *J*_1_ = 10.9 Hz, *J*_2_ = 4.3 Hz, H-19β); 3.21 (dd, 1H, *J*_1_ = 8.3 Hz, *J*_2_ = 3.7 Hz, H-3α); 4.56 (s, 1H, H-29 *pro-E*); 5.02 (s, 2H, H-30); 5.05 (s, 1H, H-29 *pro-Z*); 7.48 (m, 1H, H-37); 8.23 (td, 1H, *J*_1_ = 7.7 Hz, *J*_2_ = 2.0 Hz, H-36); 8.54 (dd, 1H, *J*_1_ = 4.9 Hz, *J*_2_ = 1.7 Hz, H-35); 8.71 (s, 1H, H-31); 9.06 (d, 1H, *J* = 2.0 Hz, H-34). ^13^C NMR (125 MHz, CDCl_3_): δ = 14.65; 15.35; 16.11; 18.24; 20.95; 26.13; 27.09; 27.96; 27.92; 29.05; 29.58; 32.05; 34.31; 36.68; 37.17; 38.11; 38.84; 40.63; 40.86; 42.39; 48.72; 50.43; 50.51; 51.43; 55.29; 56.12; 78.92; 110.77; 120.63; 128.42; 128.55; 132.13; 144.54; 146.36; 148.57; 149.58; 168.10; 178.88. MS (ESI-): *m*/*z* (%) = 599 (100, [M-H]^-^). HRMS (ESI-TOF) *m*/*z* calcd for C_37_H_52_N_4_O_3_ [M-H]^-^ 599.3956, found 599.3945. Note: deprotection procedure 2 was also tried with yield of 60% and the attempt to prepare compound **11c** from free azide **8** by the general procedure for click reaction gave the best yield of 76%.

Compound **11e** was obtained from 150 mg (0.17 mmol) of **10e** by the general deprotection procedure 1 at r.t. for 30 h. The yield of white crystals was 124 g (64%): mp 150–151°C (CH_2_Cl_2_). IR (DRIFT): 2600–3400, 1731, 1651 cm^-1^. ^1^H NMR (500 MHz, CDCl_3_): δ 0.76 (s, 1H); 0.82 (s, 3H); 0.92 (s, 3H); 0.97 (s, 6H, H-23, 24, 25, 26, 27); 2.30 (d, 1H, *J* = 9.2 Hz); 2.99 (s, 6H, H-39, 40); 2.99 (m, 1H, H-19β) 3.20 (dd, 1H, *J*_1_ = 11.2 Hz, *J*_2_ = 4.9 Hz, H-3α); 4.71 (s, 1H, H-29 *pro-E*); 4.97 (AB-system, 2H, *J*_*GEM*_ = 15.5 Hz, H-30); 5.07 (s, 1H, H-29 *pro-Z*); 6.67 (d, 2H, *J* = 9.1 Hz, H-34, 38); 7.62 (s, 1H, H-31); 7.70 (d, 2H, *J* = 8.7 Hz, H-35, 37). ^13^C NMR (125 MHz, CDCl_3_): δ = 14.67; 15.33; 16.04; 16.10; 18.23; 20.92; 26.83; 27.31; 27.96; 29.64; 31.85; 34.28; 36.55; 36.69; 37.18; 38.32; 38.68; 38.84; 40.48; 40.49; 40.66; 42.38; 43.53; 50.39; 54.48; 55.26; 78.96; 111.79; 112.52; 118.61; 118.86; 126.71; 126.90 148.43; 149.65; 150.44; 179.98. MS (ESI+): *m*/*z* (%) = 643 (100, [M+H]^+^). HRMS (ESI+) *m*/*z* calcd for C_40_H_58_N_4_O_3_ [M+H]^+^ 643.4582, found 643.4583.

Compound **11f** was obtained from 150 mg (0.16 mmol) of **10f** by the general deprotection procedure 1 at r.t. for 30 h. The yield of white crystals was 148 mg (75%): mp 141–142°C (EtOAc). IR (DRIFT): 2500–3450, 1729, 1647 cm^-1^. ^1^H NMR (500 MHz, CDCl_3_): δ 0.76 (s, 3H); 0.82 (s, 3H); 0.92 (s, 3H); 0.97 (s, 6H, H-23, 24, 25, 26, 27); 2.18 (dd, 1H, *J*_1_ = 12.5 Hz, *J*_2_ = 2.9 Hz); 2.29 (d, 1H, *J* = 12.8 Hz); 3.02 (td, 1H, *J*_1_ = 10.9 Hz, *J*_2_ = 4.6 Hz, H-19β); 3.20 (dd, 1H, *J*_1_ = 11.2 Hz, *J*_2_ = 4.7 Hz, H-3α); 4.72 (s, 1H, H-29 *pro-E*); 5.00 (AB-system, 2H, *J*_*GEM*_ = 15.8 Hz, H-30); 5.08 (s, 1H, H-29 *pro-Z*); 7.45 (d, 2H, *J* = 8.6 Hz, H-35, 37); 7.73 (s, 1H, H-31); 7.77 (d, 2H, *J* = 8.6 Hz, H-34, 38). ^13^C NMR (125 MHz, CDCl_3_): δ = 14.65; 15.33; 16.02; 16.09; 18.23; 20.92; 26.87; 27.31; 27.95; 29.63; 30.14; 31.27; 31.90; 32.00; 34.26; 34.64; 36.67; 37.15; 38.27; 38.67; 38.82; 40.63; 42.36; 43.31; 43.43; 50.37; 54.71; 55.24; 56.29; 78.95; 111.78; 119.76; 125.47; 125.72; 127.72; 147.93; 149.67; 151.29; 180.99. MS (ESI+): *m*/*z* (%) = 456 (100, [M+H]^+^), 478 (4, [M+Na]^+^). HRMS (ESI+) *m*/*z* calcd for C_42_H_61_N_3_O_3_ [M+H]^+^ 456.4786, found 456.4786. Note: deprotection procedure 2 at r.t. for 4 h was also tried with the yield of 73%.

Compound **11g** was obtained from 150 mg (0.18 mmol) of **10g** by the general deprotection procedure 1 at 50°C for 20 h. The yield of white crystals was 144 mg (81%): mp 127–129°C (CHCl_3_). IR (DRIFT): 2500–3400, 1724, 1652 cm^-1^. ^1^H NMR (500 MHz, CDCl_3_): δ 0.76 (s, 3H); 0.82 (s, 3H); 0.92 (s, 3H); 0.95 (s, 3H); 0.97 (s, 3H, H-23, 24, 25, 26, 27); 2.12 (m, 2H) 2.13 (m, 1H); 2.29 (d, 1H, *J* = 3.0 Hz); 2.97 (td, 1H, *J*_1_ = 10.9 Hz, *J*_2_ = 4.3 Hz, H-19β); 3.21 (dd, 1H, *J*_1_ = 8.3 Hz, *J*_2_ = 3.7 Hz, H-3α); 4.66 (s, 1H, H-29 *pro-E*); 4.90 (AB-system, 2H, *J*_*GEM*_ = 15.5 Hz, H-30); 5.05 (s, 1H, H-29 *pro-Z*); 7.24 (s, 1H, H-31). ^13^C NMR (125 MHz, CDCl_3_): δ = 14.66; 15.33; 16.01; 16.09; 18.20; 20.87; 25.12; 26.67; 27.31; 27.95; 29.61; 31.92; 33.24; 34.28; 36.64; 36.74; 37.16; 38.26; 38.73; 38.82; 40.63; 40.66; 42.36; 43.45; 50.16; 50.37; 54.22; 55.28; 56.29; 78.96; 111.87; 120.02; 149.66; 152.98; 180.95. MS (ESI+): *m*/*z* (%) = 592 (100, [M+H]^+^), 614 (10, [M+Na]^+^). HRMS (ESI+) *m*/*z* calcd for C_37_H_57_N_3_O_3_ [M+H]^+^ 592.4473, found 592.4474.

### General information about the biological assays

#### Cell lines

Biological assays were performed in concordance with our previous publications [29, 58; 60; 61]. All cells (if not indicated otherwise) were purchased from the American Tissue Culture Collection (ATCC). The CCRF-CEM line is derived from T lymphoblastic leukemia, evincing high chemosenzitivity, K562 represent cells from an acute myeloid leukemia patient sample with bcr-abl translocation, U2OS line is derived from osteosarcoma, HCT116 is colorectal tumor cell line and its p53 gene knock-down counterpart (HCT116p53-/-, Horizon Discovery Ltd, UK) is a model of human cancers with p53 mutation frequently associated with poor prognosis, A549 line is lung adenocarcinoma. The daunorubicin resistant subline of CCRF-CEM cells (CEM-DNR bulk) and paclitaxel resistant subline K562-TAX were selected in our laboratory by the cultivation of maternal cell lines in increasing concentrations of daunorubicine or paclitaxel, respectively. The CEM-DNR bulk cells overexpress MRP-1 and P-glycoprotein protein, while K562-TAX cells overexpress P-glycoprotein only. Both proteins belong to the family of ABC transporters and are involved in the primary and/or acquired multidrug resistance phenomenon [58]. MRC-5 and BJ cell lines were used as a non-tumor control and represent human fibroblasts. The cells were maintained in nunc/corning 80 cm^2^ plastic tissue culture flasks and cultured in cell culture medium according to ATCC or Horizon recommendations (DMEM/RPMI 1640 with 5 g/L glucose, 2 mM glutamine, 100 U/mL penicillin, 100 mg/mL streptomycin, 10% fetal calf serum, and NaHCO_3_).

#### Cytotoxic MTS assay

MTS assay was performed at Institute of Molecular and Translational Medicine by robotic platform (HighResBiosolutions). Cell suspensions were prepared and diluted according to the particular cell type and the expected target cell density (25000–35000 cells/mL based on cell growth characteristics). Cells were added by automatic pipetor (30 μL) into 384 well microtiter plates. All tested compounds were dissolved in 100% DMSO and four-fold dilutions of the intended test concentration were added in 0.15 μL aliquots at time zero to the microtiter plate wells by the echoacustic non-contact liquid handler Echo550 (Labcyte). The experiments were performed in technical duplicates and three biological replicates at least. The cells were incubated with the tested compounds for 72 h at 37°C, in a 5% CO_2_ atmosphere at 100% humidity. At the end of the incubation period, the cells were assayed by using the MTS test. Aliquots (5 μL) of the MTS stock solution were pipetted into each well and incubated for additional 1–4 h. After this incubation period, the optical density (OD) was measured at 490 nm with an Envision reader (Perkin Elmer). Tumor cell survival (TCS) was calculated by using the following equation: TCS = (OD_drug-exposed well_/mean OD_control wells_) × 100%. The IC_50_ value, the drug concentration that is lethal to 50% of the tumor cells, was calculated from the appropriate dose-response curves in Dotmatics software.

#### Cell cycle and apoptosis analysis

Suspension of CCRF-CEM cells, seeded at a density of 1.10^6^ cells/mL in 6-well panels, were cultivated with the 1 or 5 × IC_50_ of tested compound in a humidified CO_2_ incubator at 37°C in RPMI 1640 cell culture medium containing 10% fetal calf serum, 10 mM glutamine, 100 U/mL penicillin, and 100 μg/mL streptomycin. Together with the treated cells, control sample containing vehicle was harvested at the same time point after 24 h. After another 24 hours, cells were then washed with cold PBS and fixed in 70% ethanol added dropwise and stored overnight at -20°C. Afterwards, cells were washed in hypotonic citrate buffer, treated with RNAse (50 μg/mL) and stained with propidium iodide. Flow cytometer using a 488 nm single beam laser (Becton Dickinson) was used for measurement. Cell cycle was analyzed in the program ModFitLT (Verity), and apoptosis was measured in logarithmic model expressing percentage of the particles with propidium content lower than cells in G0/G1 phase (<G1) of the cell cycle. Half of the sample was used for pH3^Ser10^ antibody (Sigma) labeling and subsequent flow cytometry analysis of mitotic cells [61].

#### BrDU incorporation analysis (DNA synthesis)

For this analysis, the same procedure of cultivation as previously was used. Before harvesting, 10 μM 5-bromo-2-deoxyuridine (BrDU), was added to the cells for puls-labeling for 30 min. Cells were fixed with ice-cold 70% ethanol and stored overnight. Before the analysis, cellswere washed with PBS, and resuspended in 2 M HCl for 30 min at room temperature to denature their DNA. Following neutralization with 0.1 M Na_2_B_4_O_7_ (Borax), cells were washed with PBS containing 0.5% Tween-20 and 1% BSA. Staining with primary anti-BrDU antibody (Exbio) for 30 min at room temperature in the dark followed. Cells were than washed with PBS and stained with secondary antimouse-FITC antibody (Sigma). Cells were then washed with PBS again and incubated with propidium iodide (0.1 mg/mL) and RNAse A (0.5 mg/mL) for 1 h at room temperature in the dark and afterwards analyzed by flow cytometry using a 488 nm single beam laser (FACSCalibur, Becton Dickinson) [61].

#### BrU incorporation analysis (RNA synthesis)

Cells were cultured and treated as above. Before harvesting, pulse-labeling with 1 mM 5-bromouridine (BrU) for 30 min followed. The cells were then fixed in 1% buffered paraformaldehyde with 0.05% of NP-40 in room temperature for 15 min, and then stored in 4°C overnight. Before measurement, they werewashed in PBS with 1% glycin, washed in PBS again, and stained by primary anti-BrDU antibody crossreacting to BrU (Exbio) for 30 min at room temperature in the dark. After another washing step in PBS cells were stained by secondary antimouse-FITC antibody (Sigma). Following the staining, cells were washed with PBS and fixed with 1% PBS buffered paraformaldehyde with 0.05% of NP-40 for 1 hour. Cells were washed by PBS, incubated with propidium iodide (0.1 mg/mL) and RNAse A (0.5 mg/mL) for 1 h at room temperature in the dark, and finally analyzed by flow cytometry using a 488 nm single beam laser (FACS Calibur, Becton Dickinson) [61].

## Supporting information

S1 Fig ^1^H NMR spectrum of 3. (DOCX)

S2 Fig ^13^C NMR spectrum of 3. (DOCX)

S3 Fig ^1^H NMR spectrum of 4. (DOCX)

S4 Fig ^13^C NMR spectrum of 4. (DOCX)

S5 Fig ^1^H NMR spectrum of 5. (DOCX)

S6 Fig ^13^C NMR spectrum of 5. (DOCX)

S7 Fig ^1^H NMR spectrum of 6. (DOCX)

S8 Fig ^13^C NMR spectrum of 6. (DOCX)

S9 Fig ^1^H NMR spectrum of 7. (DOCX)

S10 Fig ^13^C NMR spectrum of 7. (DOCX)

S11 Fig ^1^H NMR spectrum of 8. (DOCX)

S12 Fig ^13^C NMR spectrum of 8. (DOCX)

S13 Fig ^1^H NMR spectrum of 9a. (DOCX)

S14 Fig ^13^C NMR spectrum of 9a. (DOCX)

S15 Fig ^1^H NMR spectrum of 9b. (DOCX)

S16 Fig ^13^C NMR spectrum of 9b. (DOCX)

S17 Fig ^1^H NMR spectrum of 9c. (DOCX)

S18 Fig ^13^C NMR spectrum of 9c. (DOCX)

S19 Fig 2D COSY NMR spectrum of 9c. (DOCX)

S20 Fig 2D ROESY NMR spectrum of 9c. (DOCX)

S21 Fig 2D HMQC NMR spectrum of 9c. (DOCX)

S22 Fig 2D HMBC NMR spectrum of 9c. (DOCX)

S23 Fig 2D ^15^N-HMBC NMR spectrum of 9c. (DOCX)

S24 Fig ^1^H NMR spectrum of 9d. (DOCX)

S25 Fig ^13^C NMR spectrum of 9d. (DOCX)

S26 Fig 2D COSY NMR spectrum of 9d. (DOCX)

S27 Fig 2D ROESY NMR spectrum of 9d. (DOCX)

S28 Fig 2D HMQC NMR spectrum of 9d. (DOCX)

S29 Fig 2D HMBC NMR spectrum of 9d. (DOCX)

S30 Fig 2D ^15^N-HMBC NMR spectrum of 9d. (DOCX)

S31 Fig ^1^H NMR spectrum of 9e. (DOCX)

S32 Fig ^13^C NMR spectrum of 9e. (DOCX)

S33 Fig ^1^H NMR spectrum of 9f. (DOCX)

S34 Fig ^13^C NMR spectrum of 9f. (DOCX)

S35 Fig ^1^H NMR spectrum of 9g. (DOCX)

S36 Fig ^13^C NMR spectrum of 9g. (DOCX)

S37 Fig ^1^H NMR spectrum of 9h. (DOCX)

S38 Fig ^13^C NMR spectrum of 9h. (DOCX)

S39 Fig ^1^H NMR spectrum of 10a. (DOCX)

S40 Fig ^13^C NMR spectrum of 10a. (DOCX)

S41 Fig ^1^H NMR spectrum of 10b. (DOCX)

S42 Fig ^13^C NMR spectrum of 10b. (DOCX)

S43 Fig ^1^H NMR spectrum of 10c. (DOCX)

S44 Fig ^13^C NMR spectrum of 10c. (DOCX)

S45 Fig ^1^H NMR spectrum of 10d. (DOCX)

S46 Fig ^13^C NMR spectrum of 10d. (DOCX)

S47 Fig ^1^H NMR spectrum of 10e. (DOCX)

S48 Fig ^13^C NMR spectrum of 10e. (DOCX)

S49 Fig 2D COSY NMR spectrum of 10e. (DOCX)

S50 Fig 2D ROESY NMR spectrum of 10e. (DOCX)

S51 Fig 2D HMQC NMR spectrum of 10e. (DOCX)

S52 Fig 2D HMBC NMR spectrum of 10e. (DOCX)

S53 Fig 2D ^15^N-HMBC NMR spectrum of 10e. (DOCX)

S54 Fig ^1^H NMR spectrum of 10f. (DOCX)

S55 Fig ^13^C NMR spectrum of 10f. (DOCX)

S56 Fig ^1^H NMR spectrum of 10g. (DOCX)

S57 Fig ^13^C NMR spectrum of 10g. (DOCX)

S58 Fig ^1^H NMR spectrum of 11a. (DOCX)

S59 Fig ^13^C NMR spectrum of 11a. (DOCX)

S60 Fig ^1^H NMR spectrum of 11b. (DOCX)

S61 Fig ^13^C NMR spectrum of 11b. (DOCX)

S62 Fig 2D COSY NMR spectrum of 11b. (DOCX)

S63 Fig 2D ROESY NMR spectrum of 11b. (DOCX)

S64 Fig 2D HMQC NMR spectrum of 11b. (DOCX)

S65 Fig 2D HMBC NMR spectrum of 11b. (DOCX)

S66 Fig 2D ^15^N-HMBC NMR spectrum of 11b. (DOCX)

S67 Fig ^1^H NMR spectrum of 11c. (DOCX)

S68 Fig ^13^C NMR spectrum of 11c. (DOCX)

S69 Fig ^1^H NMR spectrum of 11d. (DOCX)

S70 Fig ^13^C NMR spectrum of 11d. (DOCX)

S71 Fig ^1^H NMR spectrum of 11e. (DOCX)

S72 Fig ^13^C NMR spectrum of 11e. (DOCX)

S73 Fig ^1^H NMR spectrum of 11f. (DOCX)

S74 Fig ^13^C NMR spectrum of 11f. (DOCX)

S75 Fig ^1^H NMR spectrum of 11g. (DOCX)

S76 Fig ^13^C NMR spectrum of 11g. (DOCX)
