# Supplementary material for: Cytotoxic conjugates of betulinic acid and substituted triazoles prepared by Huisgen Cycloaddition from 30-azidoderivatives
Source: PLoS One. 2017 Feb 3;12(2):e0171621. doi: 10.1371/journal.pone.0171621 (PMC5291411; doi:10.1371/journal.pone.0171621)
Supplement: S6 Fig — (DOCX) [file pone.0171621.s006.docx]

**S6 Fig** ^13^C NMR spectrum of **5**.
