# Supplementary material for: Cytotoxic conjugates of betulinic acid and substituted triazoles prepared by Huisgen Cycloaddition from 30-azidoderivatives
Source: PLoS One. 2017 Feb 3;12(2):e0171621. doi: 10.1371/journal.pone.0171621 (PMC5291411; doi:10.1371/journal.pone.0171621)
Supplement: S52 Fig — (DOCX) [file pone.0171621.s052.docx]

**S52 Fig** 2D HMBC NMR spectrum of **10e**.
